# CLIC1 null mice demonstrate a role for CLIC1 in macrophage superoxide production and tissue injury

**DOI:** 10.14814/phy2.13169

**Published:** 2017-03-08

**Authors:** Barbara Ulmasov, Jonathan Bruno, Kiyoko Oshima, Yao‐Wen Cheng, Stephen P. Holly, Leslie V. Parise, Terrance M. Egan, John C. Edwards

**Affiliations:** ^1^ Department of Internal MedicineSaint Louis UniversitySt. LouisMissouri; ^2^ UNC Kidney CenterUniversity of North CarolinaChapel HillNorth Carolina; ^3^ Department of PathologyMedical College of WisconsinMilwaukeeWisconsin; ^4^ Department of Biochemistry and BiophysicsUniversity of North CarolinaChapel HillNorth Carolina; ^5^ Department of Pharmacology and PhysiologySaint Louis UniversitySt. LouisMissouri

**Keywords:** Chloride permeability, CLIC1, Ezrin, macrophage, NADPH oxidase, superoxide

## Abstract

We generated and studied CLIC1 null (C1KO) mice to investigate the physiological role of this protein. C1KO and matched wild‐type (WT) mice were studied in two models of acute toxic tissue injury. CLIC1 expression is upregulated following acute injury of WT kidney and pancreas and is absent in C1KOs. Acute tissue injury is attenuated in the C1KOs and this correlates with an absence of the rise in tissue reactive oxygen species (ROS) that is seen in WT mice. Infiltration of injured tissue by inflammatory cells was comparable between WT and C1KOs. Absence of CLIC1 increased PMA‐induced superoxide production by isolated peritoneal neutrophils but dramatically decreased PMA‐induced superoxide production by peritoneal macrophages. CLIC1 is expressed in both neutrophils and macrophages in a peripheral pattern consistent with either plasma membrane or the cortical cytoskeleton in resting cells and redistributes away from the periphery following PMA stimulation in both cell types. Absence of CLIC1 had no effect on redistribution or dephosphorylation of Ezrin/ERM cytoskeleton in macrophages. Plasma membrane chloride conductance is altered in the absence of CLIC1, but not in a way that would be expected to block superoxide production. NADPH oxidase redistributes from an intracellular compartment to the plasma membrane when WT macrophages are stimulated to produce superoxide and this redistribution fails to occur in C1KO macrophages. We conclude that the role of CLIC1 in macrophage superoxide production is to support redistribution of NADPH oxidase to the plasma membrane, and not through major effects on ERM cytoskeleton or by acting as a plasma membrane chloride channel.

## Introduction

The CLICs are a closely related family of proteins whose physiological roles in health and disease remain a matter of intense debate (Littler et al. 2010). CLICs were initially identified and described as chloride channels of intracellular compartments (Landry et al. 1993; Valenzuela et al. 1997), and several groups have demonstrated that purified recombinant CLIC proteins can confer ion permeability to phospholipid membranes (Duncan et al. 1997; Edwards et al. 1998; Tonini et al. 2000; Tulk et al. 2002; Warton et al. 2002; Berryman et al. 2004; Singh et al. 2007); however, this hypothesis has been widely challenged and definitive evidence of an endogenous CLIC clearly functioning as a channel in vivo remains elusive. CLICs have also been shown to associate with the cytoskeleton (Berryman and Bretscher 2000; Shanks et al. 2002) and a growing body of evidence indicates CLICs may function in regulation of cytoskeletal interactions and redistribution (Jiang et al. 2014; Pierchala et al. 2010; Wegner et al. 2010; Tavasoli et al. 2016). Whether as ion channels or as cytoskeletal regulators, CLICs clearly play a role in tubulogenesis of endothelial cells and of renal tubular epithelial cells (Ulmasov et al. 2009; Chou et al. 2016). CLICs show homology to the family of glutathione‐S‐transferases (Dulhunty et al. 2001) and compelling evidence indicates that at least CLIC1 functions as a specific oxidoreductase (Al Khamici et al. 2015). CLICs have been shown to redistribute to the nucleus under some circumstances where they may be involved in gene regulation (Valenzuela et al. 2000; Shukla et al. 2009, 2016; Malik et al. 2010). CLICs have been implicated in regulation of apoptosis (Suh et al. 2004, 2005). Finally, CLICs can be found in the extracellular space including as cargo in exosomes where they have been implicated in clotting and metastasis, and could serve as tumor markers (Tang et al. 2013; Macpherson et al. 2014; Gurski et al. 2015). A skeptic may conclude that these bizarrely unrelated observations coming from numerous groups around the world can hardly all be correct. A believer could suggest an intriguing hypothesis that CLICs serve as some master regulator that links oxidoreductive state of the cell and extracellular environment with organization of the cytoskeleton, membrane trafficking, ion permeability, and gene regulation. Either might suggest that the use of genetically manipulated mice could be a powerful approach to help resolve these issues.

There are six distinct *Clic* genes in mammals, with one gene (*Clic5*) subject to alternate splicing leading to two different protein products. Thus, mammals are known to express at least seven different CLIC proteins. CLIC1 has been of particular interest in that it is ubiquitously expressed, with particularly prominent expression in the apical domains of a variety of polarized epithelia including stomach, intestine, colon, intrahepatic bile ducts, pancreatic ducts, airway, and kidney proximal tubule (Ulmasov et al. 2007). It is also highly expressed in macrophages where it is upregulated in response to endotoxin (Valenzuela et al. 1997). Evidence for channel activity is arguably most robust for CLIC1. Others have implicated CLIC1 as a plasma membrane chloride channel in microglia that supports superoxide production (Milton et al. 2008; Averaimo et al. 2010), and, independently, as supporting maximal acidification of lysosomes leading to antigen processing/presentation by macrophages (Jiang et al. 2012).

Superoxide production by both macrophages and neutrophils plays a key role in numerous forms of tissue injury and is catalyzed by the enzyme NADPH oxidase (NOX2). An important difference in regulation of NOX2 activity between macrophages and neutrophils is that activation of NOX2 in macrophages requires regulated trafficking to the plasma membrane, while activation of NOX2 in neutrophils does not (Johansson et al. 1995; Casbon et al. 2012).

To try to delineate more clearly the role of CLIC1, we generated mice in which the *Clic1* gene has been inactivated. One *Clic1* null mouse has been reported previously (Qiu et al. 2010). Those mice were reported to be essentially normal except for abnormalities in platelet number and function. Here, we report characterization of independently generated *Clic1* null mice (C1KO), focusing on a possible role for CLIC1 in inflammation and response to injury. Our data support several novel conclusions. C1KO mice are essentially normal in an unstressed laboratory environment, but C1KO females are smaller than wild type and heterozygous littermates. C1KO mice generate less tissue reactive oxygen species (ROS) in two independent models of acute toxic tissue injury. Unlike wild‐type (WT) cells, C1KO macrophages fail to increase superoxide production in response to phorbol ester and this correlates with lack of redistribution of NADPH oxidase to the plasma membrane. We conclude that CLIC1 is instrumental in the local superoxide production that occurs during acute toxic tissue injury, perhaps by supporting intracellular membrane trafficking of the NADPH oxidase protein.

## Materials and Methods

### Generation of *Clic1* ^*‐/‐*^ Mice

Nucleotide coordinates are numbered from NCBI mouse genome sequences NT_039662.3. Fragments of the *Clic1* gene were amplified from mouse genomic DNA (strain 129X1/SvJ) using polymerase chain reaction (PCR). An upstream fragment (positions 1299971–1302114) from exon 1 through intron 1, and a downstream fragment (1302856–1307283) from intron 4 into intron 5 were inserted into the KpnI‐XbaI and XhoI‐NotI sites, respectively, of pNT‐Cass‐loxA (gift of Drs. S. Tomatsu and W. Sly, St. Louis University, St. Louis MO).

Embryonic stem cell transformation and screening were performed as we previously described for the *Clic4* gene (Ulmasov et al. 2009). Blastocyst injection and generation of chimeric offspring were carried out by the Transgenic Core Facility at the University of North Carolina at Chapel Hill. Animals were genotyped by PCR amplification of tail DNA. Primers for genotyping the CLIC1 lineage were 5′AGCTAGCCAAGACTTAACTGTTCCTCTGC and 5′TCCATCTCCCTGACAGCCGAGCTCACAG. Chimeric mice were separately bred with the robust outbred strain, CD‐1, and with the inbred C57/B6 strain (both from Charles River). Experiments were performed after at least seven iterations of backcrosses. pNT‐Cass‐loxA vector is designed so that the cassette containing the neomycin gene is eliminated during the first passage through the male germ line. Thus, all mice generated from the initial male chimeras contain a deleted gene with only a residual lox‐P motif at the site of the deletion. All animal work was approved by the Institutional Animal Care and Use Committees of either the University of North Carolina Chapel Hill, or St. Louis University as appropriate. Animal blood tests were performed by the Animal Core Lab at the University of North Carolina.

### Reagents

Our CLIC1‐specific polyclonal rabbit antibodies, AP823 and AP1089, have been previously described (Tulk and Edwards 1998; Ulmasov et al. 2007). Commercial antibodies were as follows: Rabbit monoclonal anti‐phospho ERM, mouse monoclonal anti‐ezrin, and HRP‐conjugated anti‐rabbit and anti‐mouse IgG antibodies were from ThermoFisher. Anti‐GAPDH and anti‐mouse F4/80 from Santa Cruz, rat anti‐mouse neutrophil (now known as LY‐6B.2 alloantigen) from ABD‐Serotec, and anti‐NADPH oxidase (Nox2/gp91phox) from Bioss. Fluorescent‐labeled secondary antibodies from Life Sciences, ABC kit for HRP immunohistochemistry from Vector Labs. Dihydroethidium and 6‐methoxy‐N‐ethylquinolinium iodide (MEQ) from ThermoFisher. Cerulein, folic acid, p‐iodonitrotetrazolium violet, butylated hydroxytoluene, thiobarbituric acid, and phorbol‐12‐myristate‐13‐acetate (PMA) from Sigma‐Aldrich.

### Pancreas injury model

Cerulein was dissolved at 135 *μ*g/ml in 100 mmol/L sodium bicarbonate pH 8.75. Stock cerulein was then diluted with normal saline to provide a dose of 50 *μ*g/kg in 100 *μ*L volume for each mouse, which was given by intraperitoneal injection hourly for six doses. Control mice received 100 *μ*L injections of isotonic saline. Mice were euthanized at 9 h after the first dose at which time blood was collected for amylase and lipase determination, and pancreases were removed for further testing.

### Pancreas histology

Formalin‐fixed pancreas chunks were embedded in paraffin, sectioned, and stained with hematoxylin and eosin. Slides were graded by an experienced pathologist masked to treatment group and using a semi‐quantitative histopathology scoring system similar to the one that we have described previously (Ulmasov et al. 2013). Pancreatic sections were graded on four criteria: vacuolization, necrosis, inflammation, edema, on the following scales. Vacuolization was graded as 0 =  absent, 1 = 5%–14%, 2 = 15%–35%, 3 = 36%–50%, and 4 =  >50%. Necrosis was graded as 0 =  absent, 1 =  periductal necrosis <5%, 2 =  focal necrosis 5%–20%, and 3 =  diffuse periductal necrosis 20%–50%. Inflammation was graded as 0 =  absence of inflammatory infiltrates, 1 =  inflammatory infiltration in ducts, 2 =  inflammatory infiltration in the parenchyma <50%, and 3 =  inflammatory infiltration in the parenchyma >50%. Edema was graded as 0 =  absent, 1 =  focally increased between lobules, 2 =  diffusely increased between lobules, and 3 =  acini disrupted and separated.

### Immunostaining

Formalin‐fixed paraffin‐embedded pancreas sections were passed through xylene and graded alcohols. Endogenous peroxidases were blocked and sections stained with AP823 antibody using the Vector ABC kit per the manufacturer's recommendations. Unfixed kidney tissue was embedded in TissueTek and quick frozen. Five micron sections were fixed on the slide with methanol and stained as previously described (Ulmasov et al. 2007). Freshly isolated neutrophils and macrophages were plated on poly‐L‐lysine‐coated glass and incubated overnight. Cells were fixed with 4% paraformaldehyde in phosphate‐buffered saline for 30 min at room temperature, blocked, permeablilized, and stained with the AP1089 antibody and cell‐specific markers as in Ulmasov et al. (2007).

### Quantitative PCR of pancreas

Isolation of total RNA from pancreatic tissue and real‐time quantitative PCR was conducted as described previously (Ulmasov et al. 2010). PCR primers were synthesized by Life Technologies (Carlsbad, CA) based on the sequences from Primer Bank (Wang and Seed 2003). Results were calculated with normalization to ribosomal protein, large, P0 (Rplp0) messenger RNA (mRNA). Rplp0 was chosen as the housekeeping control gene because it previously was shown that its mRNA does not change significantly with single or multiple episodes of cerulein‐induced pancreatitis (Gukovsky et al. 1998; Lugea et al. 2003; Perides et al. 2005). The comparative threshold cycle method (Livak and Schmittgen 2001) was used to calculate changes in mRNA abundance.

The primer sequences of transcripts evaluated by quantitative PCR are:

Rplp0: 5′‐AGATTCGGGATATGCTGTTGGC‐3, 5′‐TCGGGTCCTAGACCAGTGTTC‐3′;

Clic1: 5′‐GAAGAACAACCTCAGGTCGAAC‐3′, 5′‐CTCTGTCCGTCTCTTGGTGTC‐3′

Clic4: 5′‐TCAAGGCCGGAAGTGATGG‐3′, 5′‐TCAAGGCCGGAAGTGATGG‐3′

### Kidney injury model

Folic acid was dissolved at 50 mg/mL in 300 mmol/L sodium bicarbonate and filter sterilized. Mice were administered 0.3 mg folic acid per gm body weight via IP injection. Control mice received an equal volume injection of 300 mmol/L sodium bicarbonate. Mice treated with folic acid were euthanized at 24 and 48 h after injections at which time a blood sample was collected for BUN assay and kidneys removed for further testing.

### Quantitative PCR from kidney

Procedures were essentially identical to those used for pancreas, except primers were:

GAPDH 5′GACCCCTTCATTGACCTCAACTAC and 5′GCTCCTGGAAGATGGTGATGG

CLIC4 5′AAAGCATTGGAAACTGCCCC and 5′TTCAGGTCAACGGTTGTGACA

CLIC1 5′GAAGAACAACCTCAGGTCGAAC and 5′CTCTGTCCGTCTCTTGGTGTC.

### Bleeding times

Mice were anaesthetized with an 8 *μ*L/gm body weight intraperitoneal injection of 10 mg/mL ketamine, 0.8 mg/mL xylazine. When mice were unresponsive to foot pad pinch, 0.5 cm was cut from the tip of the tail with a sharp razor blade. The end of the tail was immediately immersed in normal saline at 37^°^C and the time to cessation of bleeding was recorded. After recovery from anesthesia, mice were returned to their cages.

### Platelet methods

Mouse blood was obtained via cardiac puncture according to Wang et al. (2009). Briefly, 0.7–0.9 mL blood was drawn into 0.7 mL acid citrate dextrose (ACD)/saline (1:4) containing 0.1 *μ*mol/L prostaglandin E_1_ (PGE_1_), gently mixed and spun at 100*g* to obtain platelet rich plasma (PRP). PRP was stored in a separate tube and the RBC fraction was washed with 0.8 mL ACD/saline (1:10) and spun again as above. PRP fractions from the same mouse were pooled and spun at 500*g* for 10 min to generate a platelet pellet. After removal of the supernatant, platelets were resuspended in 500 *μ*L warm CGS (13 mmol/L citrate, 50 mmol/L glucose, 120 mmol/L NaCl, pH 7.0) containing 1U/mL apyrase and warmed at 37°C for 15 min. Platelets were spun at 500*g* for 10 min and resuspended in modified HEPES‐Tyrodes buffer (5 mmol/L HEPES, 137 mmol/L NaCl, 2.7 mmol/L KCl, 11.9 mmol/L NaHCO_3_, 0.42 mmol/L NaH_2_PO_4_, 1 mmol/L MgCl_2_, 5.5 mmol/L glucose, 0.1% delipidated BSA, pH 7.4) and allowed to stand for 30 min to disperse clumps. During this period, platelets were counted using a Z1 Particle Counter (Coulter‐Beckman) and adjusted to 2 × 10^8^/mL in HEPES‐Tyrodes containing 1 mmol/L CaCl_2_. Aggregation rates were determined with a Chrono‐log Model 700 lumi‐aggregometer, using ADP to initiate aggregation.

### Thiobarbaturic acid reactive substances (TBARS) assay

TBARS assay was as described (Oakes and Van Der Kraak 2003). In brief, flash frozen tissue pieces (approximately 50 mg) were homogenized in 0.25 ml of PBS (135 mmol/L NaCl, 10 mmol/L NaPhosphate, pH 7.5) with 0.5 mg/ml butylated hydroxytoluene (BHT), 0.1 mmol/L phenylmethylsulfonylfluoride (PMSF), 1 mmol/L ethylene diamine tetraacetic acid (EDTA) and kept on ice. Reaction mixes were assemble with 20 *μ*L of homogenate, 20 *μ*L of 8.1% sodium dodecyl sulfate, 150 *μ*L of 20% glacial acetic acid titrated to pH 3.5, 150 *μ*L of 0.53% thiobarbituric acid in water, 60 *μ*L of water, and 1 *μ*L of 50 mg/ml BHT. A standard curve was generated with serial dilutions of tetramethoxypropane (TMP) in water from 31.6 nmol/L to 100 *μ*mol/L. Reaction mixes were incubated at 95^°^C for 1 h, cooled on ice, and extracted with 0.5 mL of n‐butanol/pyridine 15:1. Fluorescence of the upper phase was determined with excitation at 535 nm and emission at 552 nm using a Varian Cary Eclipse fluorescence spectrophotometer. Protein concentrations were determined with the BCA assay Kit (ThermoFisher).

### Cell preparation

Peritoneal neutrophils were prepared 24 h after intraperitoneal injection of casein, and peritoneal macrophages were prepared 4 days after intraperitoneal injection of thioglycollate medium per standard protocols (Luo and Dorf 1997; Zhang et al. 2008).

### Superoxide assays with cultured cells

#### Cytochrome C assay

In total, 100,000 neutrophils in 100 *μ*L of HBS (HEPES‐buffered saline: 135 mmol/L NaCl, 10 mmol/L HEPES pH 7.4, 4 mmol/L KCl, 1 mmol/L CaCl_2,_ 1 mmol/L MgCl_2_, and 5 mmol/L glucose) were mixed with 100 *μ*L of 140 *μ*mol/L ferricytochrome C with or without 100 nmol/L phorbol myristyl acetate (PMA) and/or 20 mmol/L TEMPOL in HBS in wells of a 96‐well plate which was immediately placed in a 550 nm plate reader set at 37°C and readings taken every 20 sec.

#### p‐Iodonitrotetrazolium violet assay

Mouse peritoneal macrophages from female CD1 mice were plated at 1,000,000 cells per well on a six‐well plate and allowed to attach overnight. The cells were washed with PBS and incubated for 1 h at 37^°^C in 0.5 ml INV medium (0.5 mg/mL p‐iodonitrotetrazolium violet [Sigma‐Aldrich], DMEM and 25 mmol/L HEPES pH 7.4) with either dimethylsulfoxide (DMSO) vehicle, 100 nmol/L PMA, 20 mmol/L TEMPOL, or 100 nmol/L PMA and 20 mmol/L TEMPOL. Absorbance was read at 492 nm.

#### Dihydroethidium assay

Macrophages were plated on glass Delta T dishes in 6 mm wells defined by a silicon mask. After at least 24 h in culture, the plate was mounted on the heated stage of an inverted microscope fitted with CCD camera. Images were collected with an YFP filter set. Cells were allowed to equilibrate for 10 min in HBS. The solution was changed to HBS with 10 *μ*mol/L dihydroethidium with or without 100 nmol/L PMA, and 300 units/mL superoxide dismutase (SOD). Stock solutions of additives were 5 mmol/L dihydroethidium in DMSO, 1 mmol/L PMA in DMSO, and 30,000 units/ml SOD in phosphate‐buffered saline. All solutions contained either each additive or equal volume of vehicle. Images of cells in the focal plane of the nuclei were collected at 1 min intervals for 15 min starting immediately after addition of dihydroethidium. Individual nuclei were identified and average fluorescence intensity over each nucleus determined at each time point. Fluorescence intensity of each cell was normalized to the fluorescence intensity of that cell at the first time point and the rate of rise in fluorescence determined by linear regression over the first 6 min of recording.

### Macrophage chloride permeability

Macrophages were plated on glass coverslips at 200,000 cells per well in a 24‐well plate and allowed to attach overnight. Cells were loaded with MEQ by osmotic shock by replacing medium with 400 *μ*L of 10 mmol/L MEQ in a solution of 0.5X HEPES‐buffered NaNitrate (HBNN; 1X consists of 135 mmol/L NaNO_3_, 4 mmol/L KNO_3_, 1 mmol/L calcium gluconate, 1 mmol/L magnesium gluconate, 1 mmol/L sodium phosphate dibasic, and 5 mmol/L glucose) for 10 min at 37^°^C. The solution was aspirated and replaced with 1X HBNN. The coverslip was mounted on an inverted epifluorescence microscope fitted with a CCD camera and a superfusion system. The sample was superfused with 1X HBNN. In samples treated with IAA‐94, the drug was added to 50 *μ*mol/L at this time and remained present through the remainder of the recording. After 15 min, solution was changed to HBNN with 100 nmol/L PMA (and IAA‐94, as appropriate) for those samples that were treated with PMA. Five minutes later, perfusion solution was changed to HBS, with or without PMA and/or IAA‐94 as appropriate. Images were collected at 5 sec intervals and converted to a stack. Areas of interest were defined over individual cells and fluorescence intensity for each cell determined from each image in the stack over the initial 25 sec after substitution of chloride for nitrate. Fluorescence intensity of each cell over time was normalized to the fluorescence intensity of that cell in the first image. The rate of fractional quenching was taken as a measure of the plasma membrane anion permeability.

### Statistical methods

Significance of differences between experimental mean values was determined by standard T‐testing or analysis of variance as appropriate, using software embedded in Microsoft Excel, and online at Social Science Statistics (http://www.socscistatistics.com/pvalues/tdistribution.aspx). The significance of difference between median BUN values following kidney injury was determined using the Rank‐Sum test for non‐normally distributed data sets.

## Results

### Disruption of *Clic1* gene

**Figure 1:**
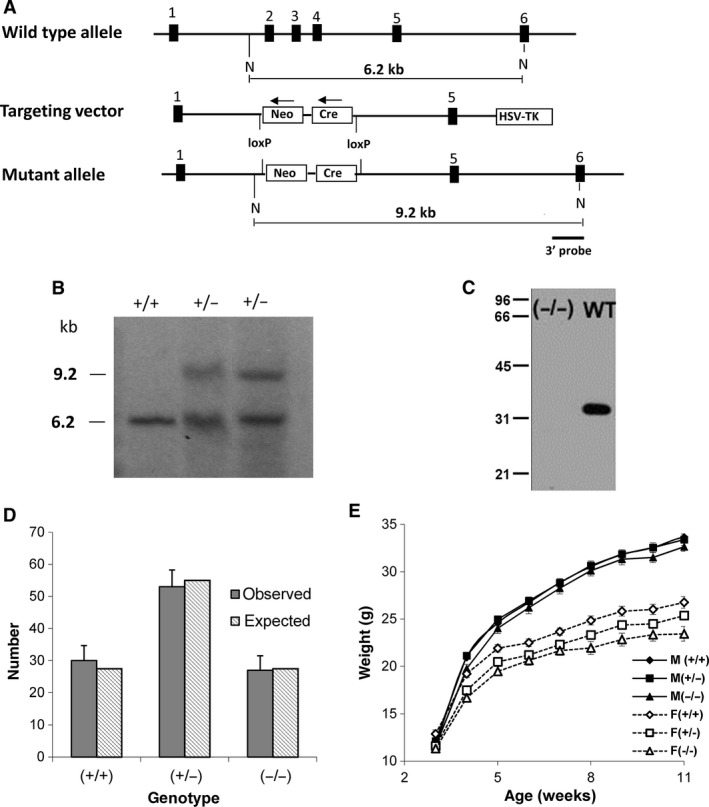
Generation and characterization of CLIC1 null mice. (A) Maps of the WT allele, the targeting vector, and the predicted allele resulting from homologous recombination are shown. Numbered exons are designated by thick bars. N indicates NheI restriction sites and the size of NheI restriction fragments are shown. (B) Southern blot of NheI‐digested genomic DNA from one WT and two heterozygous homologous recombinant ES cell lines, probed with the 3′ probe designated in panel A, showing the 6.2 kB WT fragment and the 9.2 kB recombinant fragment. (C) Western blot of 50 *μ*g total kidney protein from Clic1 (‐/‐) or WT mice as labeled, probed with CLIC1 specific antibody AP1089. Migration positions of molecular weight standards (in kD) are marked. (D) Number of observed offspring of each genotype in 110 consecutive pups from crosses between CLIC1 heterozygotes (dark bars). Error bar represents standard error. The expected Mendelian ratio of 0.25:0.5:0.25 is plotted for comparison (light bars). (E) Growth curves for the 110 pups. Males: filled symbols; Females: open symbols; WT: diamonds; Heterozygotes: squares; KO: triangles. There is no difference in weight among the male offspring. The KO females are significantly smaller than the WT females at all time points, with the Het females having an intermediate average weight.

A map of the *Clic1* gene, targeting vector, and structure of the recombined chromosomal DNA are shown in Figure 1A. This construct eliminates exons 2, 3, and 4 in the recombined product. Splicing from exon 1 to exon 5 would put exon 5 out of frame and introduce a stop codon 3 bases downstream. If the resulting message is stable, the disrupted *Clic1* gene would encode a 1649 MW protein that consists of the N‐terminal 13 amino acids of CLIC1 encoded by exon 1, followed by 1 amino acid encoded by the second reading of exon 5.

The targeting vector was used to generate stably transfected mouse embryonic stem cells. Homologous recombination was confirmed by Southern blotting of NheI digested genomic DNA as shown in Figure 1B. Recombinant stem cells were used to generate chimeric mice which were bred with outbred CD‐1 mice to generate true heterozygotes. These mice were repeatedly outbred to CD‐1 mice or separately back‐crossed to C57/B6 mice, each at least seven iterations. Resulting inbred and outbred lines were used to generate the mice used in the experiments as noted.

**Figure 2:**
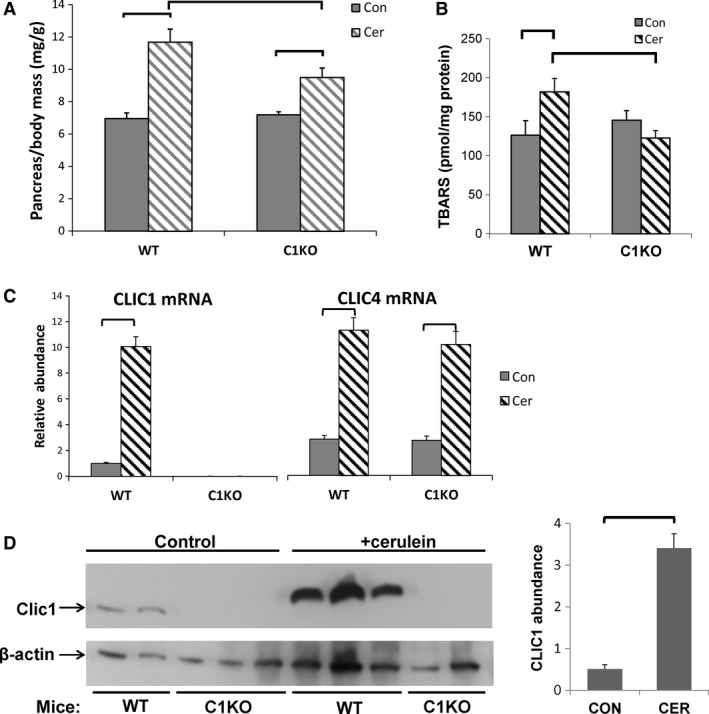
Acute pancreatitis (see also Table 2). (A) Pancreas to body mass ratio (mg/gm, *n* = 10 for each group), (B) tissue TBARS (pmole/mg protein, *n* = 9 for controls, *n* = 10 for cerulein‐treated groups), and (C) relative mRNA levels (normalized to GAPDH) in control (solid bars) and cerulein‐treated (hatched bars) WT or C1KO mice as marked. For all control groups, *n* = 9; for all cerulean‐treated groups, *n* = 10. (D) Left: Western blot of total pancreatic protein probed for CLIC1 (AP1089 antibody, upper panel) or *β*‐actin (lower panel) from control or cerulein‐treated WT or C1KO mice as marked. Right: Average pancreatic CLIC1 abundance, normalized to *β*‐actin from control or cerulean‐treated WT mice (con *n* = 2; cer *n* = 3). Data presented are means +/‐ SEM. Brackets indicate pairwise comparisons for which *P* < 0.05.

**Figure 3:**
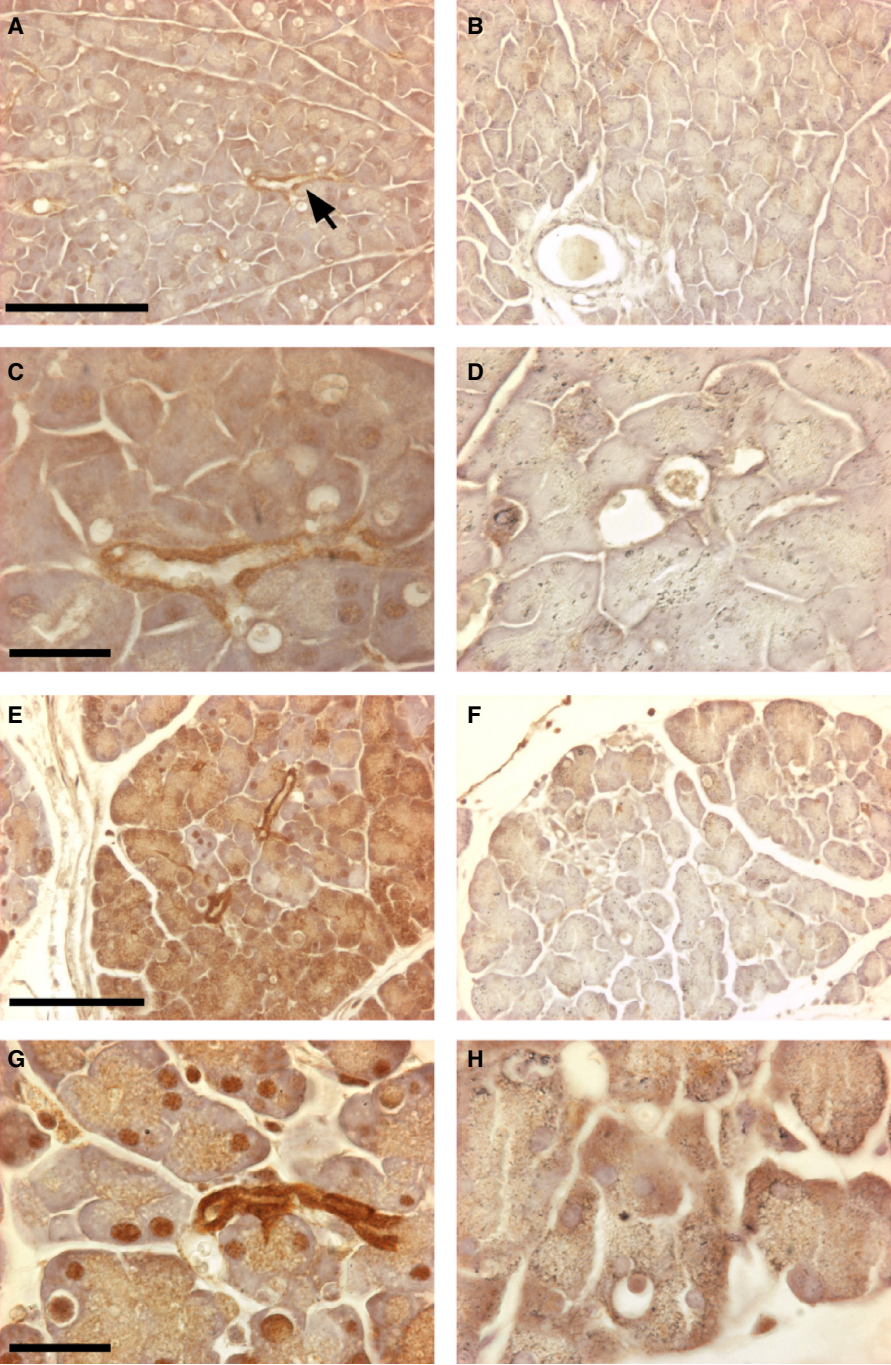
Immunostaining for CLIC1 in control and cerulein‐treated pancreas. Sections of pancreas from control (upper four panels) and cerulein‐treated (lower four panels) from WT (left column) and C1KO (right column) mice are shown. Immune reaction product is dark brown. A, B: WT (A) and C1KO (B) control pancreas at low power. Arrow denotes CLIC1 immunostained small pancreatic duct in A. Scale bar represents 100 microns. C,D: WT (C) and C1KO (D) control pancreas at high power. Notice moderate staining of small pancreatic duct and faint nuclear staining in C. Scale bar represents 25 microns. E, F: WT (E) and C1KO (F) cerulein‐injured pancreas at low power. Scale bar represents 100 microns. G, H: WT (G) and C1KO (H) cerulein‐injured pancreas at high power. Scale bar represents 25 microns. Note intensely stained pancreatic duct epithelium, patchy acinar cell cytoplasmic, and nuclear staining in E and G.

**Figure 4:**
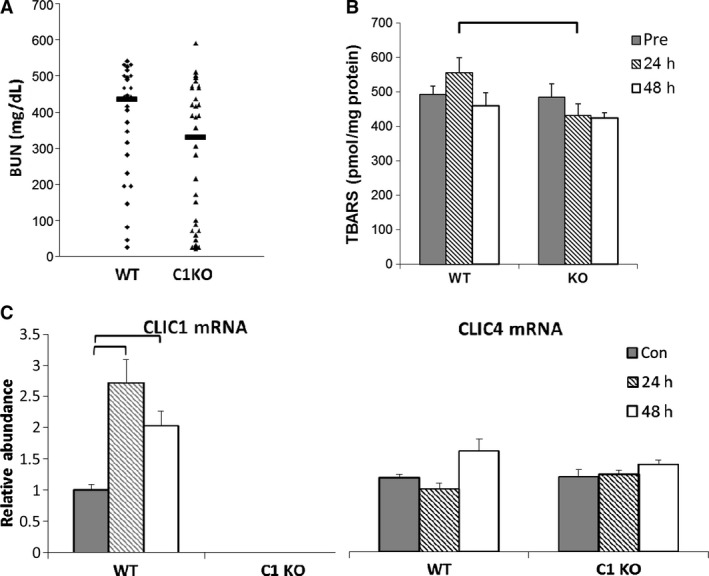
Acute kidney injury (see also Table 3). (A) Serum BUN (mg/dl) at 48 h post‐folic acid injection from WT and C1KO mice. Value from each animal designated by small symbols, median of each group shown with bar. (B) Tissue TBARS (pmole/mg protein). See Table 3 for statistical values for panels A and B. (C) Relative mRNA levels (normalized to GAPDH) in control (solid bars) and folic acid treated mice at 24 h (hatched bars) or 48 h (open bars) after folic acid injection in WT and C1KO mice. N values for WT samples at 0, 24, and 48 h after folic acid injury equal 8, 8, and 6, respectively. *N* values for C1KO samples at 0, 24, and 48 h equal 9, 9, and 8, respectively. Data presented in B and C are means +/‐ SEM. Brackets indicate pairwise comparisons for which *P* < 0.05.

**Figure 5:**
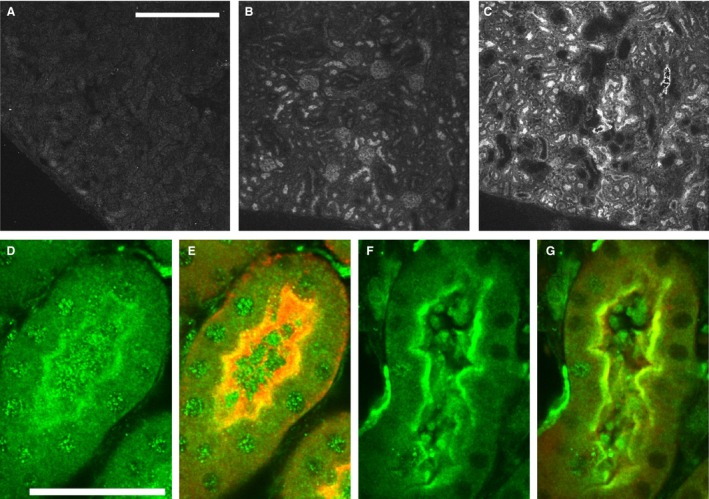
Immunofluorescent localization of CLIC1 in kidney. A–C: Low‐power images from renal cortex immunostained for CLIC1 with AP1089 antibody and imaged identically. Scale bar in A represents 400 microns and applies to panels A–C. (A) C1KO control mouse. (B) WT control mouse. The most prominent staining is in the brush border of proximal tubule. Glomeruli are also stained. (C) WT mouse 48 h after folic acid injury. CLIC1 staining is increased and is brightest in proximal tubule. Many dilated tubule segments are visible. (D–G) High‐power images of kidneys from WT mice. Scale bar in D represents 50 microns and applies to panels D–G. (D) WT control kidney showing CLIC1 stain. (E) Same field as in D, showing CLIC1 stain (green) and LTA lectin (red) marking proximal tubule brush border. Notice diffuse CLIC1 staining of brush border and speckled staining of proximal tubule nuclei. (F) WT mouse kidney 48 h after folic acid injury showing CLIC1 stain. (G) Same field as in F, showing CLIC1 stain (green) and LTA lectin (red). Staining has concentrated in the apical pole of the proximal tubule, nuclear staining has largely disappeared.

Animals were genotyped by PCR amplification of tail DNA with primers that would yield a 1010 bp product from the WT gene and a 275 bp product from the recombinant. Absence of CLIC1 in Clic1(‐/‐) mice was confirmed by RT‐PCR (see Figs. 2 and 4), western blotting (Fig. 1C), and immunostaining (see Figs. 3 and 5).

Non‐sibling Clic1(+/‐) mice in CD‐1 background were mated and 110 consecutive pups were genotyped, yielding 30 wild type, 53 heterozygote, and 27 Clic1 null mice (Fig. 1D). Clic1 null mice are neither over‐ nor under‐represented in the offspring, indicating there is no pre‐natal mortality associated with the absence of CLIC1. These littermates were monitored for growth and development. There was no significant difference in weight, rate of growth, behavior or appearance between CLIC1 WT, het, or null male mice (Fig. 1E). However, C1KO female mice are significantly smaller than the WT mice already at 3 weeks of age and the difference remains significant through 3 months (at 11 weeks of age, WT 26.8 ± 0.62 gm, *n* = 19; CLIC1 null 23.4 ± 0.77 gm, *n* = 10, *P* = 0.003). Female heterozygotes are intermediate in size between the WT and C1KO throughout the growth curve, indicating a dose effect of CLIC1 on growth in females only. Standard blood and urine chemistries were performed on cohorts of young adults 6–12 weeks old. No systematic differences in serum electrolytes, hemoglobin, urine chemistries, urine volume, or total urine protein were found among the groups of mice. Standard screening histology of a wide range of organs did not identify any structural abnormalities. No differences in behavior, spontaneous activity, or gait were noted. The mice appear to have a normal life−span. Both male and female C1KO mice were fertile and litter size was normal.

### Platelet function

A previous description of a different C1KO mouse reported defective platelet function with prolonged bleeding times and altered platelet counts (Qiu et al. 2010). Using mice in the C57/B6 background, we analyzed platelet count and function with standard bleeding times and an *ex vivo* aggregation assay as shown in Table 1. We found no difference in bleeding times or platelet aggregation in response to ADP between our C1KO and wild‐type mice. No difference in platelet count was confirmed in the CD‐1 genetic background as well (not shown).

**Table 1:** Platelet count and function in WT and CLIC1 null mice

	Wild Type	C1KO	*P* value
Platelet Count (X10^3^/*μ*L)	556 ± 64, *n* = 10	564 ± 22, *n* = 12	(NS)
Bleeding time (sec)	166 ± 37, *n* = 11	148 ± 18, *n* = 12	(NS)
Platelet aggregation: % aggregation 2.5 min after agonist			
3.3 *μ*mol/L ADP	68.7 ± 5.8, *n* = 3	69.0 ± 1.4, *n* = 4	(NS)
0.33 *μ*mol/L ADP	40.3 ± 10, *n* = 3	36.0 ± 4.3, *n* = 4	(NS)

Values are mean ± SEM.

### CLIC1 in response to injury

Since CLIC1 has been implicated in superoxide production by macrophages and microglia, and since superoxide is thought to play an important role in many models of acute tissue injury, we used our C1KO mice to look for a potential role for CLIC1 in two models of tissue injury in which superoxide production is thought to play a role, acute pancreatitis and acute toxic kidney injury.

### Acute pancreatitis

We used a cerulein model of pancreatitis (Kim 2008; Lerch and Gorelick 2013). To induce acute injury, equal numbers of male and female mice aged 6–9 weeks of each genotype were given an intraperitoneal injection of cerulean every hour for six doses. Nine hours after the first dose, mice were sacrificed and the pancreas was excised and weighed. Samples were taken for histochemical staining, RNA isolation, total protein extraction, and thiobarbituric acid reactive substance (TBARS) assay as a surrogate for superoxide production. Blood was collected for amylase and lipase assay. This experiment was performed once.

Response to acute pancreatic injury is summarized in Table 2 and Figures 2, 3. Following acute injury, serum levels of amylase and lipase both rose dramatically and there was no difference in the serum levels of these markers of acute injury between WT and C1KO mice. Histologic assessment of acute injury and inflammation using a standard scoring scheme was not different between the WT and C1KO mice. Pancreatic weight was significantly increased following acute injury, reflecting edema, and inflammation (Fig. 2A). The increase in weight was significantly less in the C1KO than the wild‐type mice, indicating less acute edema following injury although this difference was not apparent by standard histology. Tissue level of thiobarbituric acid reactive substances (TBARS), which reflects reactive oxygen species (ROS) including superoxide, was increased following acute injury in the wild‐type mice but not in the C1KO mice (Fig. 2B).

**Table 2:** Acute pancreatic injury

	Wild type	C1KO	*P* value
Amylase			
Control	924 ± 65, *n* = 9	1174 ± 77, *n* = 8	NS
Injured	6539 ± 824, *n* = 10	7206 ± 680, *n* = 10	NS
Lipase			
Control	1055 ± 96, *n* = 9	1274 ± 245, *n* = 8	NS
Injured	9640 ± 1032, *n* = 10	10,758 ± 1432, *n* = 10	NS
Pancreas mass/mouse mass (mg/g)			
Control	69.7 ± 3.3, *n* = 10	71.8 ± 1.9, *n* = 10	NS
Injured	117.0 ± 8.0, *n* = 10	95.1 ± 5.9, *n* = 10	0.007
Injury score			
Control	0.83 ± 0.54, *n* = 6	0.71 ± 0.36, *n* = 7	NS
Injured	5.9 ± 0.46, *n* = 10	6.4 ± 0.42, *n* = 10	NS
TBARs (pmole/mg protein)			
Control	126.3 ± 18.6, *n* = 9	145.5 ± 12.2, *n* = 9	NS
Injured	182.0 ± 17.3, *n* = 10	122.9 ± 9.2, *n* = 10	0.0063

Data are means ± SEM. *P* values calculated using ANOVA.

mRNA encoding CLIC1 was upregulated 10.3‐fold during acute pancreatitis in the wild‐type mice and was undetectable in the C1KO mice (Fig. 2C). In contrast, CLIC4 mRNA was present at comparable levels in WT and C1KO mice, and increased about fourfold following injury in both WT and C1KO animals. Increased CLIC1 expression was confirmed by western blotting for a subset of the animals (Fig. 2D). Immunolocalization of CLIC1 in control wild‐type pancreas (Fig. 3A, C) showed expression primarily in the pancreatic ducts, as previously reported (Ulmasov et al. 2007), plus some low level diffuse expression in the acinar cells including the nuclei of some acinar cells, which has not been previously noted. Following injury of WT (Fig. 3E,G), CLIC1 expression was dramatically increased in the duct cells. Furthermore, there were patchy areas in which acinar cytoplasmic and/or nuclear expression was much more prominent. All these signals were absent in comparable samples from C1KO mice (Fig. 3B, D, F, and H) and with staining of pancreas from WT mice using irrelevant rabbit polyclonal antibody (not shown).

The interstitial inflammatory infiltrate was evaluated as part of the semi‐quantitative histology assessment noted above. Both WT and C1KO pancreas had minimal inflammatory cell infiltrate in the absence of injury and impressive accumulation of cells following acute injury. There was no difference noted between the WT and C1KOs. A large majority of the inflammatory infiltrate is comprised of neutrophils in both WT and C1KOs.

In summary, in this model of acute pancreatitis, CLIC1 is dramatically upregulated at the level of RNA and protein in the pancreas following acute injury, with increased protein abundance most prominent in the pancreatic duct cells but detectable increase in acinar cells as well. WT and C1KO mice had a similar level of acute pancreatic cell injury as reflected by serum amylase and lipase and by semi‐quantitative histologic scoring, but had less acute inflammation/edema as reflected in pancreatic weight, and the rise in pancreatic TBARS reflecting ROS production seen in the WT mice was absent in the C1KOs.

### Acute kidney injury

In a second model of acute injury, we assessed acute kidney injury in response to intraperitoneal injection of folic acid as previously described (Edwards et al. 2014). Age‐ and sex‐matched cohorts of WT and C1KO mice in C57/B6 background were subjected to folic acid injury and the degree of renal dysfunction assessed with serum blood urea nitrogen level at 48 h. Since, as noted previously, the severity of kidney injury in this model is not normally distributed (Edwards et al. 2014), non‐parametric methods were used to analyze significance of difference in response (Table 3 and Fig. 4A). Median urea concentration was significantly less in the C1KO mice (*P* = 0.032 using the Rank‐sum test) indicating less acute renal dysfunction.

**Table 3:** Acute kidney injury

	Wild Type	C1KO	*P* value
BUN (mg/dl)			
Control (mean)	26.2 ± 0.7, *n* = 38	25.0 ± 0.7, *n* = 43	NS
48 h post‐injury (median)	435, *n* = 29	330, *n* = 34	0.032
TBARS (pmole/mg protein)			
Control	492 ± 25, *n* = 8	485 ± 38, *n* = 9	NS
24 h post‐injury	554 ± 45, *n* = 8	431 ± 35, *n* = 8	0.0023
48 h post‐injury	459 ± 38, *n* = 6	424 ± 16, *n* = 7	NS

Data are mean ± SEM, *P* values determined by ANOVA, except for 48 h post‐injury BUNs, in which data are medians, and P determined by Rank‐Sum test for non‐normally distributed data.

A smaller group of 10‐ to 13‐week‐old mice with equal numbers of males and females of each genotype were subjected to injury, euthanized at 24 or 48 h and kidneys removed for TBARS assay, quantitative RT‐PCR, and immunostaining. This experiment was performed once. Whole kidney TBARS were measured at 0, 24, and 48 h after folic acid injection (Table 3 and Fig. 4B). TBARS showed a strong trend to increase at 24 h following injury in WT mice, but this difference did not quite reach significance at the 95% confidence interval in this data set (*P* = 0.057). TBARS did not rise in C1KO mice at 24 h after injury. Comparing TBARS level between WT and C1KO kidneys, there was no difference at baseline, but the difference in TBARs at 24 h after injury was significant (*P* = 0.0023). By 48 h, there was again no difference in TBARs between WT and C1KO kidneys. Thus, the generation of kidney TBARs (as an indicator of ROS) at 24 h following acute folic acid injury is greater in WT than in C1KO mice.

Following acute injury, *Clic1* mRNA in whole kidney increased by 2.8‐fold at 24 h and remained elevated at 48 h (Fig. 4C). *Clic1* mRNA was undetectable in C1KO kidneys. Increased CLIC1 expression at the protein level following folic‐acid‐induced acute kidney injury has been previously demonstrated (Edwards et al. 2014) and we did not attempt to reproduce that observation here. In contrast to the acute pancreatitis model, *Clic4* mRNA was not upregulated following acute kidney injury. Immunolocalization (Fig. 5) of CLIC1 in uninjured WT mice (Fig. 5B, D, and E) was predominant in the apical domain and brush border of proximal tubule cells, as previously reported (Tulk and Edwards 1998). Some proximal tubule cells showed speckled nuclear staining. There were also variable levels of expression in peritubular endothelial cells. All these signals were absent in C1KO mice (Fig. 5A). Forty‐eight hours following acute injury of WT mice (Fig. 5C, F, and G), CLIC1 immunostaining was markedly more intense and more highly polarized to the apical membrane in the injured proximal tubules in which the brush border had been lost. Nuclear staining was no longer detectable. Peritubular capillary staining was not notably altered. These changes in CLIC1 staining pattern were less prominent but qualitatively similar at 24 h post‐injury (not shown).

Midline longitudinal sections of mouse kidney before and 24 h after acute injury with folic acid were stained with markers for either macrophages (F4/80) or neutrophils (rat anti‐mouse neutrophils). Cells per high power field averaged over the entire cut surface of the kidney cortex were determined. Twenty‐four hours after folic acid injury, the number of neutrophils rose about 10‐fold while the number of macrophages was unchanged. There was no difference in the cellular infiltrates between WT and C1KO mice.

To summarize these experiments, acute kidney injury by folic acid results in a significant rise in kidney *Clic1* mRNA that is absent in the C1KO mice. The BUN level at 48 h following injury is significantly lower in the C1KO mice compared to WT, suggesting attenuation of intensity of acute injury. TBARS tend to rise in the kidney at 24 h following acute folic acid nephrotoxicity in WT mice and this rise is absent in the C1KOs; TBARS are higher in WT than C1KO kidneys at 24 h after injury. There was no difference in the interstitial infiltrate at 24 h following injury, suggesting that the difference in TBARS is due to changes in cellular rates of ROS production rather than a difference in the number of inflammatory cells present.

### Superoxide production by isolated inflammatory cells in culture

Others have suggested that CLIC1 supports superoxide production in microglia, a macrophage‐like cell, by serving as a short‐circuiting chloride conductance in the macrophage plasma membrane (Milton et al. 2008; Averaimo et al. 2010). To investigate whether superoxide production is altered in peripheral inflammatory cells, we isolated mouse peritoneal macrophages and neutrophils and assessed superoxide production in response to stimulation with phorbol ester (PMA).

**Figure 6:**
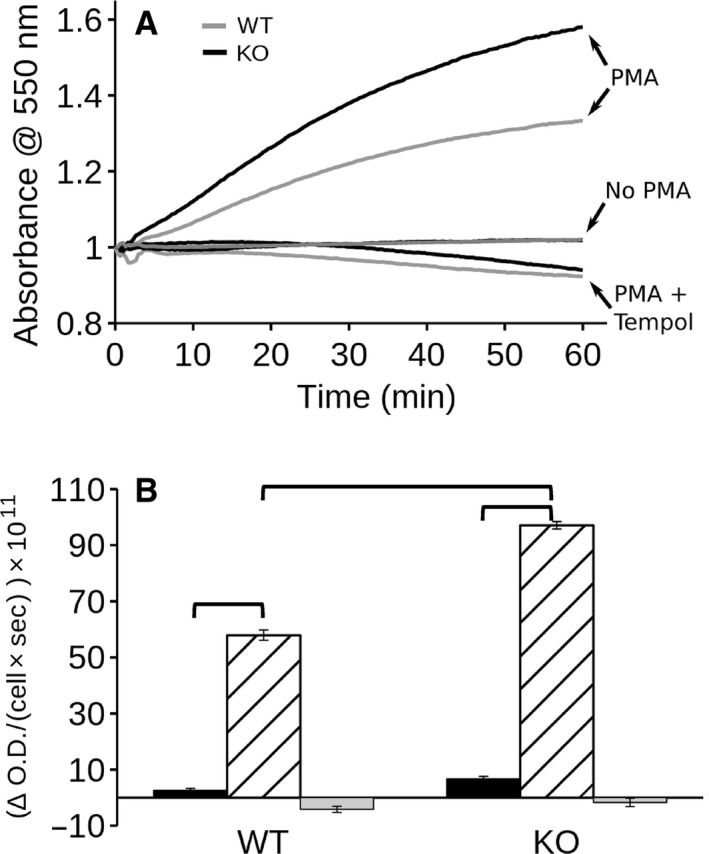
Superoxide production by neutrophils: cytochrome C assay. (A) Time course of reduction of cytochrome C by neutrophils. Traces from WT cells are shown in grey line, C1KO cells in black. Traces are from untreated cells (no PMA), cells treated with 100 nmol/L PMA (+PMA) or cells treated with PMA in the presence of Tempol (PMA + Tempol) as marked. The two “no PMA” traces lie on top of each other. (B) Rates derived by linear regression from the initial 20 min of the time course shown in panel A. Data from WT on the left, C1KO on the right as indicated. Black bar: control cells; hatched bar: PMA‐treated cells; grey bar: PMA + Tempol‐treated cells. Data are mean ± SEM. *N* = 3 for each group. Brackets indicate pairwise comparisons for which *P* < 0.05.

Peritoneal neutrophils and macrophages were isolated by standard methods. Greater than 99% of each preparation consisted of the desired cell type by immunostaining with standard cell surface markers. Superoxide production by neutrophils was assayed using a cytochrome C assay. Superoxide reduces cytochrome C to ferrocytochrome C which absorbs light at 550 nm. Tempol is a superoxide scavenger that in excess will prevent reduction of cytochrome C by superoxide. Thus, the rate of Tempol‐inhibited reduction of cytochrome C reflects the rate of superoxide production. A time course of the appearance of reduced cytochrome C is shown in Figure 6A and the initial rates derived from this time course are shown in Figure 6B. Unstimulated neutrophils showed little superoxide production and there was no significant difference in basal superoxide production between WT and C1KO neutrophils. Neutrophil superoxide generation was dramatically increased immediately following stimulation with PMA. C1KO neutrophils showed a significantly higher rate of PMA‐induced superoxide production than WT neutrophils. This result was confirmed in three independent preparations of neutrophils. Thus, absence of CLIC1 leads to enhanced PMA‐stimulated superoxide production by neutrophils.

**Figure 7:**
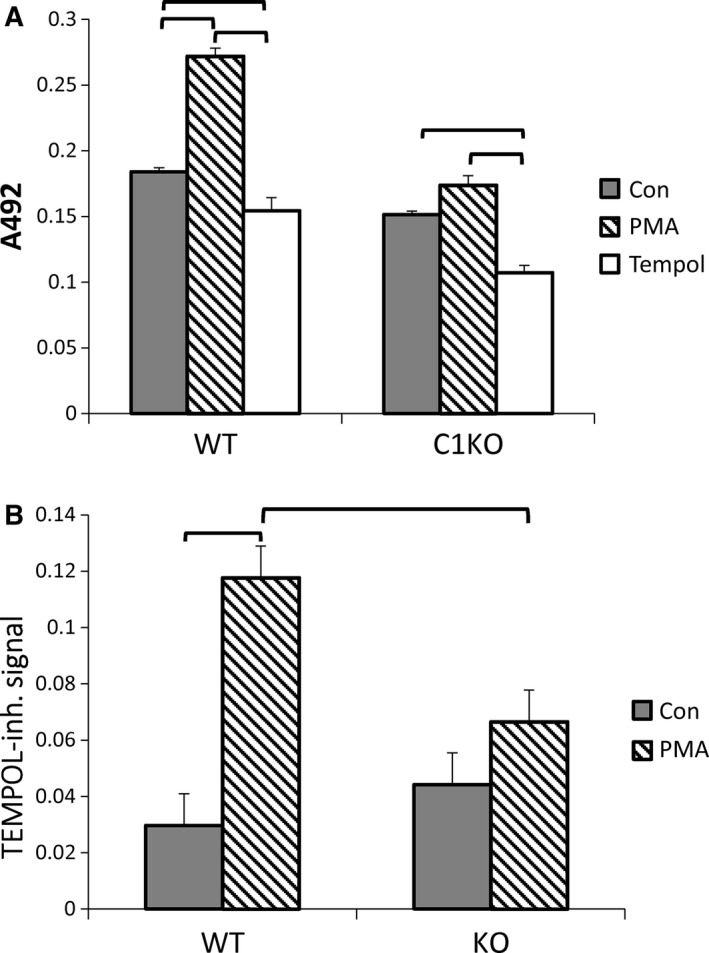
Iodonitrotetrazolium violet assay of superoxide production by macrophages. (A) Reduction of iodonitrotetrazolium violet is reflected by absorbance at 492 nm. Assay was conducted with unstimulated cells (filled bars), cells treated with 100 pm PMA (hatched bars), or PMA in the presence of Tempol (open bars) from WT or C1KO mice as indicated. (B) Tempol‐inhibited rise in A_492_ representing superoxide production. In both A and B, data shown are means ± SEM, brackets indicate pairwise comparisons for which *P* < 0.05, *n* = 3 for each group.

Superoxide production by peritoneal macrophages was much lower than that by neutrophils, and was undetectable by the cytochrome C assay. As a more sensitive assay, we first turned to an iodonitrotetrazolium violet (INTV) assay (Podczasy and Wei 1988). INTV is reduced by superoxide, yielding a colored product detectable by optical absorbance. Macrophages showed Tempol‐inhibited INTV reduction which was significantly stimulated by PMA in the WT cells (Fig. 7). The unstimulated rate of superoxide production was not different between WT and C1KO cells, but there was no significant PMA stimulation of superoxide production in the C1KOs. Thus, in contrast to neutrophils, the absence of CLIC1 decreases PMA‐stimulated superoxide production by macrophages.

**Figure 8:**
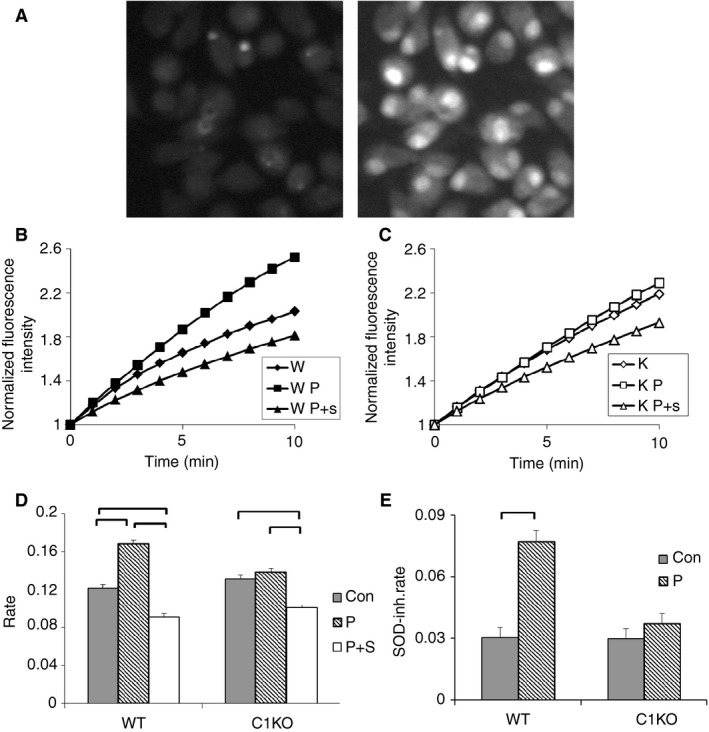
Dihydroethidium assay of superoxide production by macrophages (also see Table 4). (A) Representative microscope field of PMA‐stimulated WT macrophages at zero time (left) and 10 min (right) after initiation of recording, demonstrating accumulation of reduced dihydroethidium in nuclei. (B) Time course of nuclear fluorescence from WT cells. For each time point, the average fluorescence intensity signal from each nucleus was normalized to the fluorescence intensity of the first time point for that nucleus and the entire population averaged. Diamonds: unstimulated; squares: PMA stimulated; triangles: PMA stimulated in the presence of superoxide dismutase (SOD). Standard error bars for each point are smaller than the symbols. (C) As in B except with C1KO cells. (D) Average initial rates of change in normalized fluorescence intensity derived by linear regression of data from each individual cell from the first 6 min of recording. Filled bars: unstimulated; hatched bars: PMA stimulated; open bars: PMA stimulated in the presence of SOD. Data from WT and C1KO cells as marked. (E) SOD‐inhibited dihydroethidium reduction (arbitrary values) derived from data in D. Filled bars: unstimulated; hatched bars: PMA stimulated. In panels D and E, data are means, error bars represent SEM, brackets indicate pairwise comparisons for which *P* < 0.05.

As a more sensitive and specific assay of superoxide production in macrophages, we used fluorescence‐microscopy‐based dihydroethidium assay (Bindokas et al. 1996; Abramov and Duchen 2005; Milton et al. 2008). Reduction of dihydroethidium by superoxide results in production of a fluorescent product which intercalates into DNA; increasing nuclear fluorescence reflects production of superoxide. As a control, an excess of the superoxide scavenger superoxide dismutase (SOD) will eliminate superoxide and consequent reduction of dihydroethidium, blocking the increasing fluorescence. Thus, the SOD‐inhibitable increase in nuclear fluorescence reflects superoxide production. Macrophages attached to glass coverslip chambers were mounted on a temperature controlled epifluorescence microscope fitted with a CCD camera. Dihydroethidium was added with or without PMA and/or SOD. Images from the focal plane of the nuclei were collected at 1 min intervals over 15 min. Images from a single field at time zero and at 10 min demonstrate typical increase in nuclear fluorescence (Fig. 8A; See Table 4 for statistical values). Data were pooled from two independent sets of WT and C1KO macrophage preparations. More than 400 randomly chosen cells of each group were analyzed. Fluorescence was normalized to the initial fluorescence intensity of each cell. Average normalized fluorescence over time is shown in Figure 8B. Rate of rise in normalized fluorescence intensity over the first 6 min of recording was determined for each cell and averaged to derive mean rates for each population Results are shown in Figure 8C. As expected, WT macrophages demonstrate PMA‐inducible, SOD‐inhibitable superoxide production. The basal superoxide production is similar between WT and C1KO cells, but the PMA‐stimulated superoxide production is dramatically decreased in macrophages from C1KO mice.

**Table 4:** Rate of superoxide production (arbitrary values) by dihydroethidium assay

	Control	PMA	PMA + SOD	*P* value
Normalized rate of change in nuclear fluorescence				
WT	0.1215 ± 0.0036, *n* = 416	0.1683 ± 0.0043, *n* = 468	0.0912 ± 0.0033, *n* = 468	
C1KO	0.1312 ± 0.0039, *n* = 468	0.1384 ± 0.0039, *n* = 468	0.1012 ± 0.0030, *n* = 468	
SOD‐inhibited rate				
WT	0.0303 ± 0.0049	0.0770 ± 0.0054		0.0006
C1KO	0.0210 ± 0.0049	0.0372 ± 0.0049		NS

Data are mean ± SEM.

### Immunolocalization of CLIC1 in neutrophils

**Figure 9:**
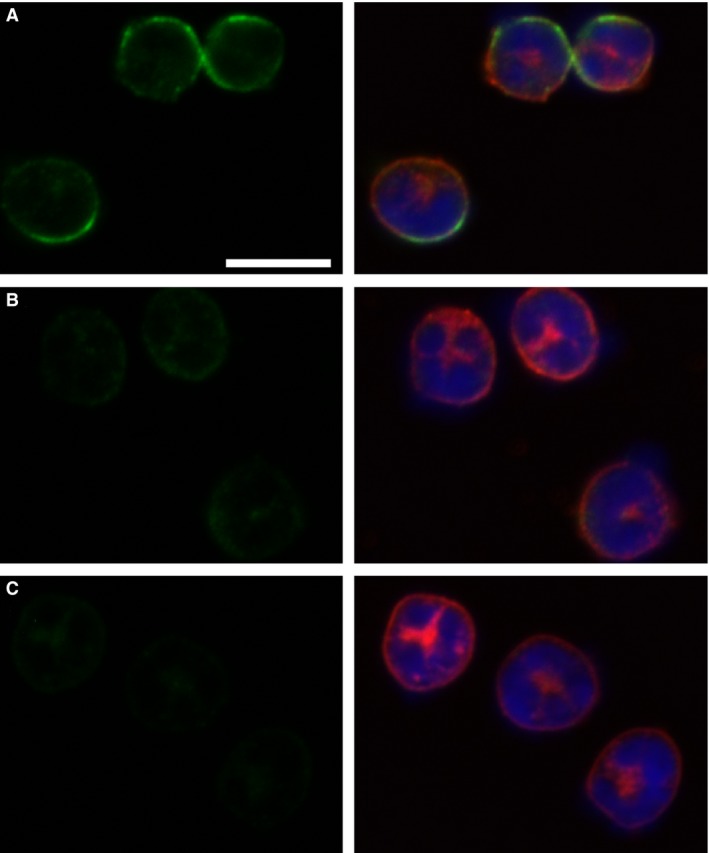
Immunolocalization of CLIC1 in neutrophils. Panels with pairs of images are shown, with the CLIC1 staining (AP823 antibody, green) only in the left column, and a merged image of CLIC1 (green), neutrophil marker (red) and nuclei (blue) in the right column. (A) Resting WT neutrophils. (B) WT Neutrophils after 10 min of stimulation with 100 nmol/L PMA. (C) Resting C1KO neutrophils. The scale bar in panel A represents 10 microns and applies to all panels.

Peritoneal neutrophils were fixed, permeabilized, and stained with antibodies against CLIC1 as well as a neutrophil‐specific cell surface marker (rat anti‐mouse neutrophil), shown in Figure 9A–D. CLIC1 is predominantly present in the periphery of the cell and redistributes away from the cell surface after stimulation with PMA.

### Immunolocalization of CLIC1 in macrophages

**Figure 10:**
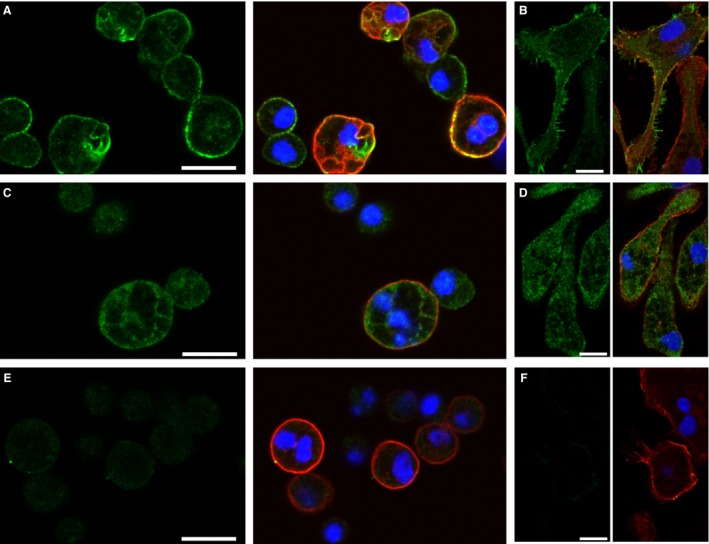
Immunolocalization of CLIC1 in macrophages. Panels with pairs of images are shown with CLIC1 staining (green) alone on the right and a merged image showing CLIC1 staining (AP823 antibody, green), macrophage marker (red), and nuclei (blue). A, B: Resting WT macrophages. Images from the domes of rounded cells in panel A, images from just above the plane of the culture surface of spread cells shown in panel B, demonstrating intense labeling of filopodia. B, C: WT macrophages after 10 min stimulation with 100 nmol/L PMA. Note redistribution from the periphery in both the domes of rounded cells (B) and the filopodia of spread cells (C). D, E: Resting C1KO macrophages showing absence of CLIC1 labeling in both the domes (D) and filopodia (E). The scale bars represent 10 microns in each image.

Primary cultures of peritoneal macrophages were grown on glass coverslip chambers, fixed and stained with antibodies against CLIC1 along with macrophage‐specific surface marker (F4/80), shown in Figure 10. CLIC1 is present throughout the cytoplasm in unstimulated macrophages, and is it prominently enriched in a peripheral distribution, consistent with a plasma membrane or peripheral cytoskeletal location (Fig. 10A). In a focal plane immediately above the culture surface, CLIC1 in some cells is present in fine cellular extensions consistent with filopodia (Fig. 10B). Following PMA stimulation (which triggers superoxide production), CLIC1 redistributes away from the peripheral location and filopodia (Fig. 10C,D). No CLIC1 staining is seen in C1KO macrophages (Fig. 10E,F).

**Figure 11:**
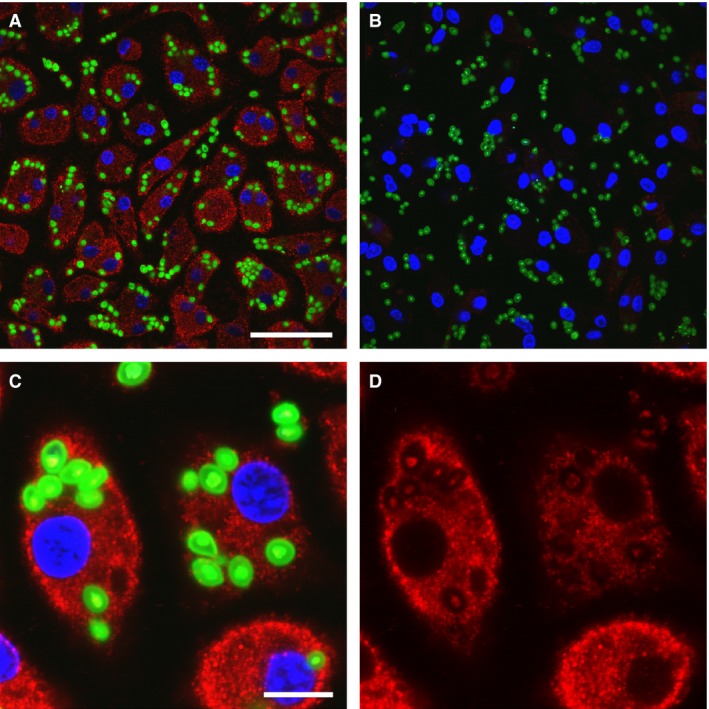
Immulocalization of CLIC1 in macrophages after phagocytosis of FITC‐labeled zymosan. CLIC1 immunolocalization with AP1089 antibody shown in red, zymosan in green. (A) WT cells. Scale bar represents 50 microns. (B) C1KO cells. Scale as in A. (C) WT cells at higher power. Scale bar represents 10 microns. (D) Same field as in C, showing only CLIC1. Note absence of concentration of CLIC1 in the phagosomal membranes surrounding the zymosan particles.

We do not find staining of limiting membranes of intracellular vesicles in resting or PMA stimulated cells. To identify phagosomes definitively, macrophages were allowed to endocytose FITC‐labeled zymosan for 30 min, then fixed and stained with antibodies to CLIC1 (Fig. 11). Zymosan endocytosis is comparable between WT and CLIC1 null macrophages, and there is no staining of zymosan‐containing phagosomes by CLIC1 antibody. Of note, the peripheral staining pattern is no longer clearly visible following phagocytosis of zymosan.

### CLIC1 and the ERM‐associated cytoskeleton

**Figure 12:**
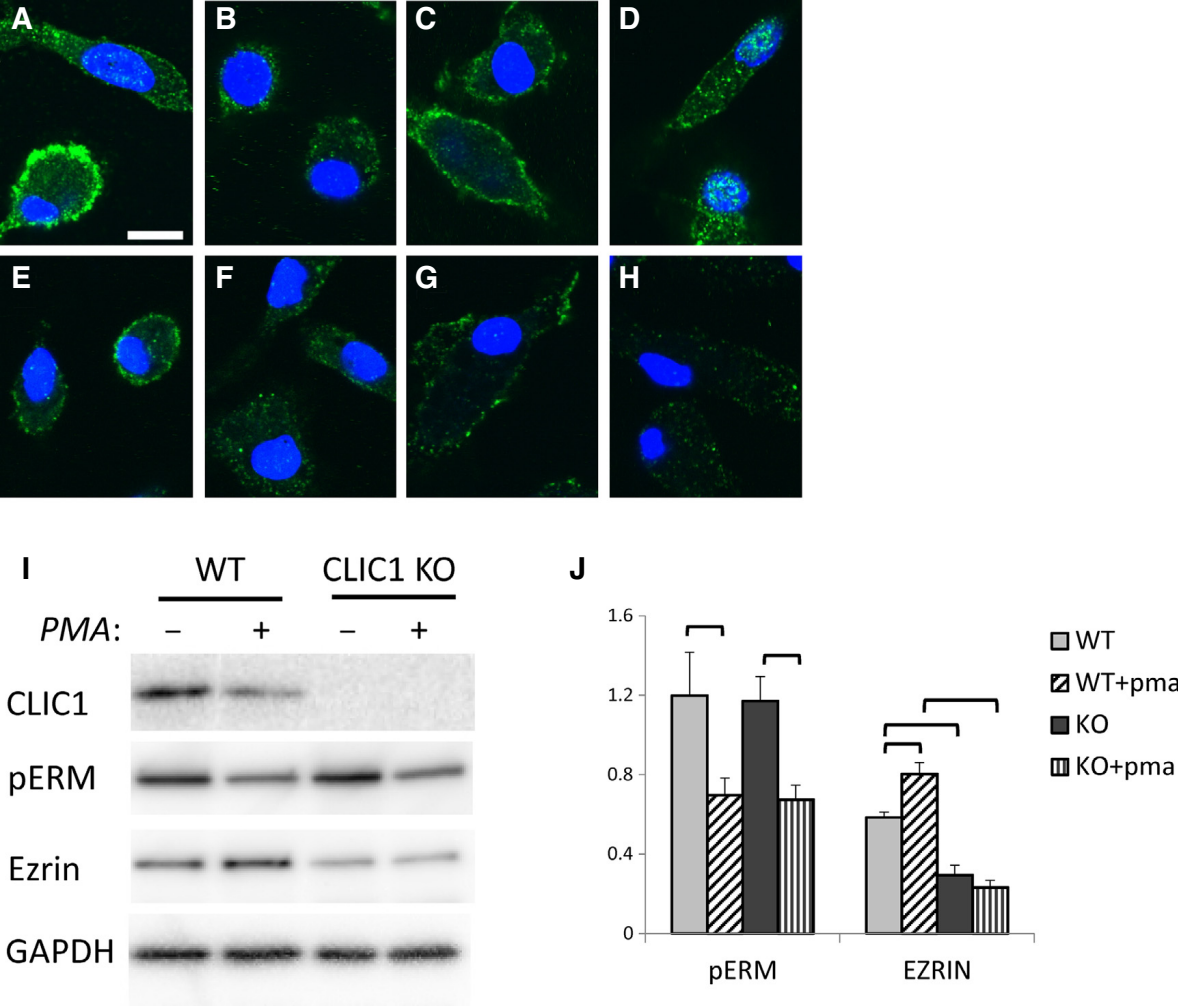
Ezrin and pERM in WT and CLIC1 null Macrophages. A–D: Macrophages stained for Ezrin (green), counterstained with DAPI to show nuclei (blue). Scale bar in A represents 10 microns and applies to panels A–H. (A) Unstimulated WT cells. (B) WT cells after 10 min exposure to PMA. (C) C1KO cells. D. C1KO cells after 10 min exposure to PMA. E‐H: Macrophages stained for pERM (green) and nuclei (blue). (E) Unstimulated WT cells. (F) WT cells after 10 min exposure to PMA. (G) C1KO cells. (H) C1KO cells after 10 min exposure to PMA. (I) Representative western blots of total cell extracts from WT or C1KO cells without or with 10 min exposure to PMA, as indicated. Blots were stripped and reprobed for each protein as indicated. (J) Western blots as in I were performed in triplicate and signal intensities quantified and normalized to the GAPDH signal. Average normalized Ezrin and pERM signals (arbitrary units) and SEM are plotted, *n* = 3 for each group. Brackets denote pairwise comparisons for which *P* < 0.05.

Since the peripheral and filopodial enrichment of CLIC1 staining is suggestive of peripheral cytoskeletal distribution, and since CLICs have been shown to associate with and alter cytoskeleton in other cell types, we examined cytoskeletal markers and their redistribution in WT and C1KO macrophages (Fig. 12). Total Ezrin (Fig. 12A–D) and phospho‐Ezrin/Radixin/Moesin (pERM) (Fig. 12E–H) both are present in a peripheral distribution in macrophages and in the basal pseudopodia. Like CLIC1, Ezrin and pERM both redistribute away from the cell periphery in response to PMA, indicating stimulation of superoxide production with PMA is accompanied by a large‐scale redistribution of the cytoskeleton. However, this redistribution is comparable in WT and C1KO cells, indicating that the presence of CLIC1 is not critical to cytoskeletal redistribution in response to PMA. To look at cytoskeletal regulation more directly, control and PMA‐treated macrophages in culture were solubilized in SDS, and probed for pERM and total Ezrin by western blotting. Results are shown in Figure 12I. Signals were normalized to GAPDH used as a loading control. Absence of CLIC1 results in a decrease in steady state total Ezrin level in macrophages. However, the level of pERM is comparable between WT and C1KO cells. Phosphorylation of ERM is decreased by about 50% following 10 min of PMA treatment in WT cells and the pERM signal falls by comparable amount in the C1KO cells. Thus, absence of CLIC1 may alter the overall expression level of Ezrin, but has no effect on total pERM level in resting cells or following PMA, indicating that an effect of CLIC1 on regulation of phosphorylation of ERM components of the cytoskeleton is likely not the mechanism by which CLIC1 alters superoxide production.

### CLIC1 and macrophage plasma membrane chloride permeability

**Figure 13:**
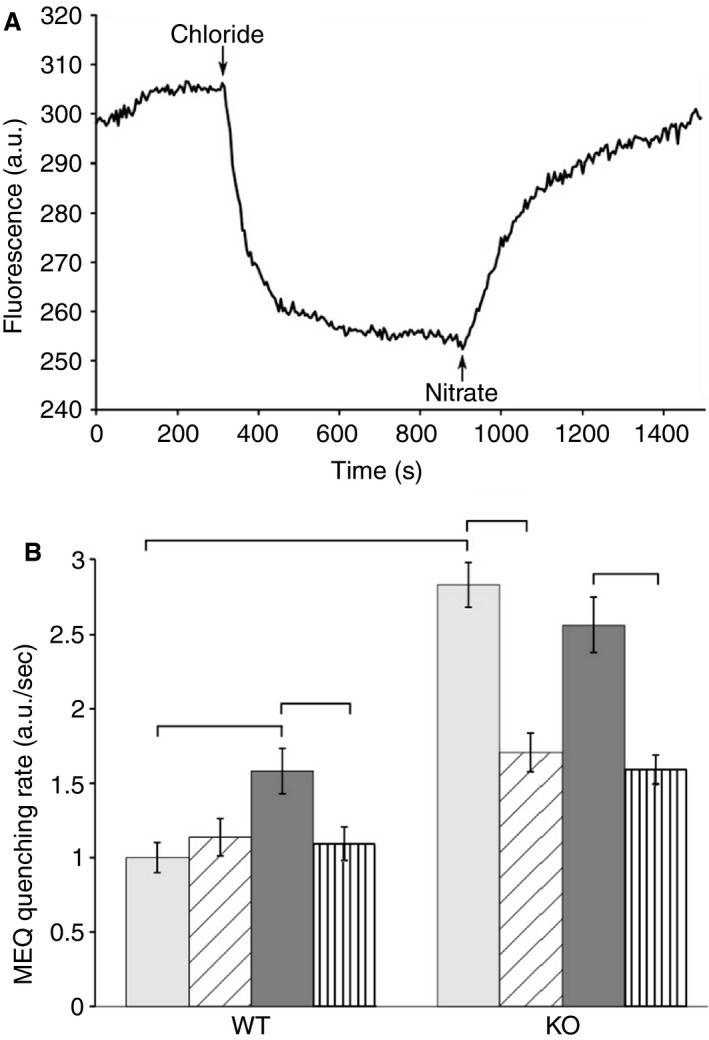
Macrophage Anion Permeability. (A) Example of fluorescence intensity recording from single MEQ‐loaded cell. Recording starts in the presence of nitrate‐containing bath solution, which is changed to chloride containing bath and then back to the nitrate at the arrows as indicated. The fractional rate of drop in fluorescence in the first 25 sec after introduction of chloride is taken as the anion permeability of the cell. (B) Average rates of fractional fluorescence quenching derived from the first 25 sec after introduction of chloride into the bath. Pale bars: unstimulated cells; diagonally hatched bars: unstimulated cells in the presence of IAA‐94; dark filled bars: PMA‐stimulated cells; vertically hatched bars: PMA stimulated cells in the presence of IAA‐94. Data are means; error bars represent SEM. See text for values, standard errors, and sample size of each group. Brackets indicate pairwise comparisons for which *P* < 0.05.

**Figure 14:**
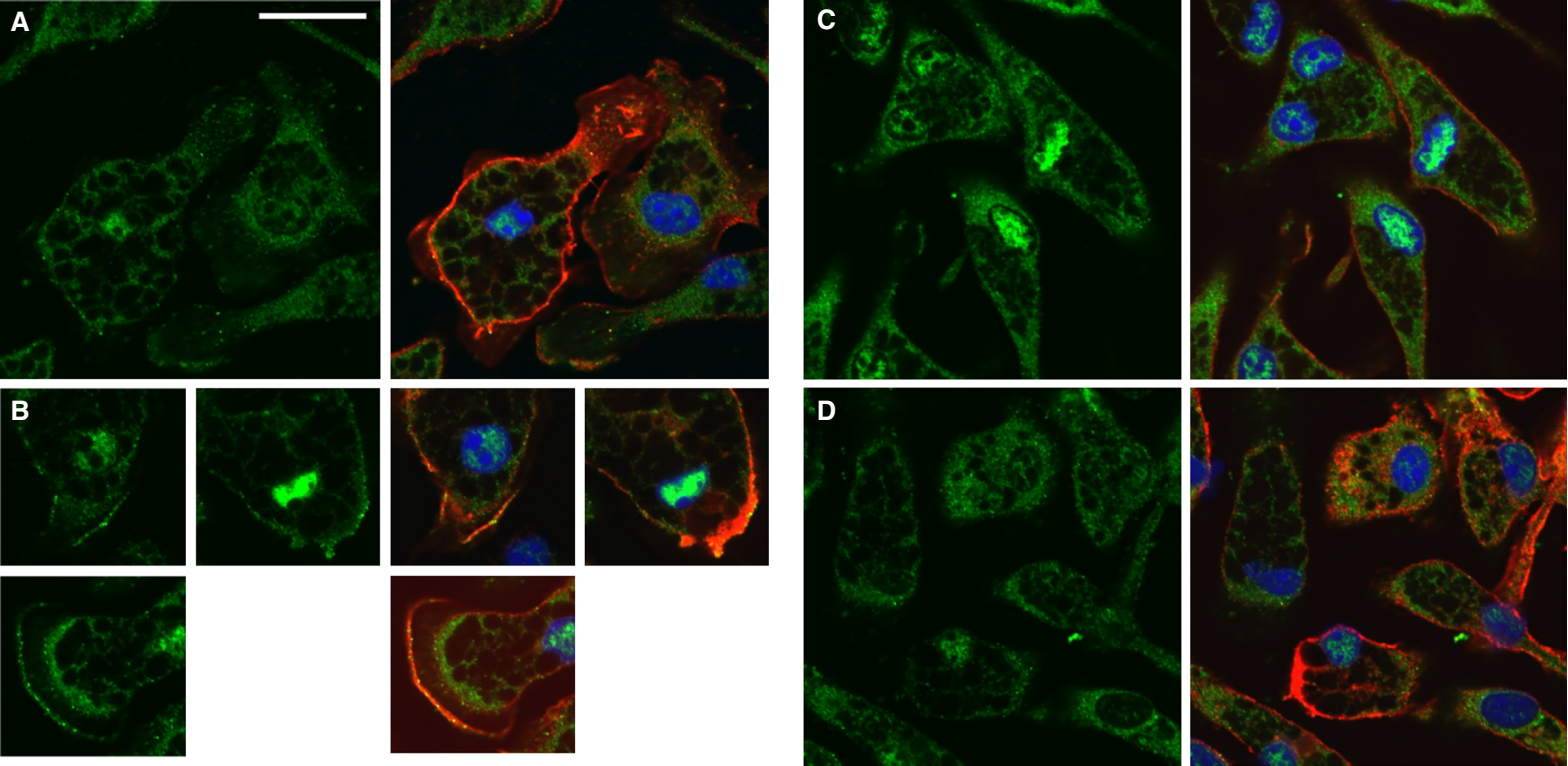
CLIC1‐dependent Redistribution of NADPH Oxidase in Macrophages. Macrophages were stained for NOX2 (green) and counterstained for the cell surface macrophage marker, F4/80 (red). A pair of images are shown for each, left image showing only the green channel, right image showing merged red and green channels. The scale bar in A represents 20 microns and applies to all panels. (A) Unstimulated WT macrophages. (B) WT macrophages after 10 min stimulation with PMA. Several individual cells are shown. (C) Unstimulated CLIC1 null macrophages. (D) CLIC1 null macrophages after 10 min exposure to PMA.

Others have suggested that CLIC1 supports superoxide production in microglia, a macrophage‐like cell, by serving as the plasma membrane chloride conductance providing a short circuit for the electrogenic superoxide production by NADPH oxidase (Milton et al. 2008). We investigated whether a similar relationship occurs in peritoneal macrophages. Plasma membrane chloride conductance of peritoneal macrophages was assessed using an MEQ assay (Biwersi and Verkman 1991), which uses the fact that MEQ fluorescence is quenched by chloride but not by nitrate, while both chloride and nitrate can permeate most cellular chloride channels. Freshly isolated macrophages were allowed to attach to glass coverslips overnight. Cells were loaded with MEQ by hypotonic shock and extracellular MEQ washed away. Loaded cells were mounted on an inverted fluorescence microscope fitted with a perfusion system. Cells were equilibrated in a Ringer's‐like solution in which all chloride was substituted with nitrate, unquenching MEQ fluorescence. While fluorescence of cells was continuously monitored, the solution was rapidly exchanged to a chloride‐containing Ringers. As chloride exchanges for intracellular nitrate through plasma membrane anion channels, the MEQ fluorescence is quenched. At the end of each experiment, chloride was again removed and replaced with nitrate, with the return of fluorescence intensity confirming that MEQ had not leaked out of the cells. Experiments were performed with wild‐type and C1KO macrophages under control conditions or after stimulation of superoxide production with 100 pM PMA, either in the presence of absence of the CLIC family inhibitor, IAA‐94. Results were pooled from two sets of paired (WT and C1KO) independent preparations of macrophages, each set assayed on different days. At least 250 cells were analyzed for each condition. Results are shown in Figure 13. A tracing from a single cell is shown in Figure 14A. The fractional rate of drop in fluorescence intensity over 25 sec immediately after introduction of chloride was determined for each individual cell and averaged to reflect the plasma membrane chloride permeability. To allow pooling of data from different days, the individual rates were normalized to the average unstimulated rate from control WT cells from that day. Average normalized rates are shown in Figure 13B. As expected, resting WT macrophages have little anion permeability (normalized rate: 1.00 ± 0.01, *n* = 202), and the baseline permeability is not inhibited by IAA‐94 (1.14 ± 0.13, *n* = 207). PMA stimulates anion permeability significantly (1.52 ± 0.15, *n* = 200) and this stimulation is blocked by IAA‐94 (1.09 ± 0.11, *n* = 219). In contrast, resting C1KO macrophages have much higher anion permeability (2.83 ± 0.15, *n* = 232) than wild‐type cells, and this permeability is substantially inhibited by IAA‐94 (1.71 ± 0.13, *n* = 230). In further contrast to WT cells, PMA does not stimulate additional activation of permeability (2.56 ± 0.18, *n* = 203) or alter extent of IAA inhibition (1.59 ± 0.10, *n* = 255) in C1KO cells. In summary, absence of CLIC1 has a dramatic effect on plasma membrane anion permeability of peritoneal macrophages. The data suggest CLIC1 suppresses IAA‐inhibitable anion permeability, and that this inhibition is released in response to PMA. Although this activity is inhibited by IAA‐94, it cannot be due to CLIC1 itself since the activity is not absent, but rather enhanced in C1KO cells. Furthermore, it is clear that superoxide production in C1KO is not limited by the absence of plasma membrane chloride permeability which is higher, not lower, in the absence of CLIC1.

### CLIC1 and NADPH oxidase redistribution

Superoxide production by macrophages is catalyzed by the NOX2 isoform of NADPH oxidase (Grimm et al. 2013; Panday et al. 2015). In resting cells, NOX2 resides in intracellular membranes. Upon stimulation, NOX2 is delivered to the plasma membrane and this redistribution is thought to be instrumental in activation of the enzyme (Johansson et al. 1995; Casbon et al. 2012). We assessed subcellular distribution of NOX2 in WT and matched C1KO macrophages (Fig. 14). Resting WT and C1KO macrophages show immunostaining for NOX2 in an intracellular pattern that does not co‐localize with a cell surface marker (Fig. 14A and C). As has been shown by others, after 10 min of stimulation of WT cells with 100 nmol/L PMA, we find a subtle but characteristic redistribution with a small fraction of the total cellular NOX2 assuming a peripheral pattern that co‐localizes with the cell surface marker (Fig. 14B). After 30 min in PMA, the cell surface distribution has disappeared (not shown). In contrast, we do not find the plasma membrane distribution of NOX2 in the C1KO cells (Fig. 14D). This observation was made in two independent matched sets of C1KO and WT macrophages. Thus, in the absence of CLIC1, NOX2 fails to move to the plasma membrane in response to PMA, and this may be the cause of lack of PMA‐stimulated superoxide production by C1KO macrophages.

## Discussion

A remarkably diverse set of functions has been attributed to CLICs in general and CLIC1 in particular. To try to establish more clearly the possible roles for CLIC1, we generated and characterized CLIC1 null mice. The data presented here using these mice support several novel conclusions. First, despite nearly ubiquitous expression, and particularly high level expression in numerous epithelia, it is clear that CLIC1 is not essential for morphogenesis, development, or reproduction, at least in an unstressed laboratory environment. Second, CLIC1 appears to play a role in at least two distinct models of acute toxic injury, with the absence of CLIC1 resulting in less acute injury. Third, in both models of acute injury, the local generation of TBARS following toxic exposure was dramatically attenuated in the absence of CLIC1, suggesting that CLIC1 is instrumental in the production of ROS in these contexts. Fourth, peritoneal macrophages from CLIC1 null mice have decreased generation of superoxide in response to PMA, while CLIC1 null peritoneal neutrophils show increased superoxide production. Fifth, absence of CLIC1 paradoxically leads to increased plasma membrane chloride permeability in resting macrophages, but also to loss of the further induction of chloride permeability following PMA stimulation that is seen in WT cells; lack of plasma membrane chloride permeability is not the cause of decreased superoxide production in C1KO macrophages. Finally, NOX2 fails to redistribute to the macrophage plasma membranes in absence of CLIC1, potentially explaining the loss of PMA‐stimulated superoxide production in C1KO macrophages.

### Lack of non‐redundant essential role for CLIC1

The absence of any obvious phenotype in unstressed C1KO mice is consistent with the absence of any human or animal disease mapped to mutations within CLIC1 to date. One of two conclusions must be true – either CLIC1 does not play a critical role in unchallenged animals or that there is functional redundancy with other proteins, perhaps other CLICs, that are expressed in the critically involved cells. The growth data suggest a sex‐specific effect of absence of CLIC1. Only the female C1KO are smaller than littermate controls, and the intermediate size of the heterozygotes suggests a gene dosage effect. The basis for this difference is unknown to us.

There is one previous report of a CLIC1 null mouse (Qiu et al. 2010). However, the details of the constructs are not identical. We deleted introns 2–4 while Qiu et al. deleted exons 1–4. Consequently, our construct would be capable of expressing a peptide consisting of the first 13 amino acids of CLIC1, whereas Qui et al. construct has no intact endogenous translational start site at all. Both our mice and Qui et al.'s mice have no detectable CLIC1 protein expression. The previous report focused on an observed difference in platelet count and platelet function which we did not find in our mice. Although a cause for this difference is not obvious, it is notable that the genetic background of our mice and those reported previously are different. We used the C57B6 background for the bleeding time and platelet aggregation experiments, while the prior report used the 129XVsvJ strain.

### CLIC1 and acute injury

We examined acute pancreatitis induced by cerulein, and acute kidney injury induced by folic acid. Both models revealed some common features in the C1KO mice compared to controls. CLIC1 was prominently upregulated in the WT and was completely absent in the C1KOs. In both tissues, the primary site of expression of CLIC1 in the uninjured wild type was in ductal epithelial cells (pancreatic duct cells in pancreas and proximal tubule cells in kidney) and the most prominent site of increased expression following injury was in those same cell types. Both models showed some modest evidence for attenuation of acute injury intensity, and most notably, in both models, the C1KO mice failed to show an increase in tissue TBARs (reflecting ROS) following injury that was prominent in the WTs. Equal numbers of male and female mice were used in the acute injury experiments, and in both models, there was no significant difference in responses to injury between males and females in either WT or C1KO mice. Each model demonstrated unique features as well.

### Acute pancreatic injury

Cerulein is a peptide analog of cholecystokinin. Prolonged supramaximal stimulation with cerulein results in acute pancreatitis with acinar cell injury and death, release of pancreatic enzymes into the circulation, and infiltration of the pancreas with inflammatory cells. The pathophysiology of cerulein induced acute pancreatitis is complex and not fully understood. However, it seems likely that the initial injury is due to excessive stimulation of acinar cells driving both overproduction of digestive enzymes and decreased pancreatic secretion into the gut, resulting in local release of activated pancreatic enzymes into the interstitium and circulation. The injured acinar cells release cytokines and superoxide that induce local injury and attract inflammatory cells which then greatly amplify injury through higher level production of cytokines and superoxide. Macrophages and neutrophils are both thought to play roles in driving cerulean‐induced pancreatic inflammation, but the cell type responsible for superoxide production has not been unequivocally identified.

Our results indicate the initial events in cerulein‐driven injury including acinar cell hypersecretion resulting in pancreatic enzyme leak into the circulation, and the subsequent recruitment of inflammatory cells is not different between WT and C1KO mice. However, the production of superoxide and the acute edema is significantly decreased in the C1KO mice, suggesting that the subsequent actions of the recruited inflammatory cells are blunted.

A unique finding in the pancreatic injury model is the increased expression of CLIC1 in the acinar cells following acute injury. Most striking is the patchy prominent staining for CLIC1 in acinar cell nuclei. CLICs are known to be targeted to the nucleus under certain circumstances where they may play a role in regulating gene expression. Perhaps the most fully characterized example is the role of CLIC4 in potentiating TGF‐*β* signaling in keratinocytes (Shukla et al. 2009, 2016). Nuclear localization of CLICs has also been implicated in driving apoptosis in some cultured cell models (Suh et al. 2004, 2005). Acinar cells surely play a role in the response to pancreatic injury through alterations of gene expression, and acinar cell apoptosis is an important mechanism by which acute injury results in pancreatic scarring and failure. The attenuation of acute swelling in the C1KO, and the observed increased expression and nuclear targeting of CLIC1 following injury in the WT suggest that CLIC1 could be altering response to injury in acinar cells as well as in ductal cells and infiltrating inflammatory cells.

### Acute kidney injury

The folic acid model of nephrotoxicity is widely used. Excessive doses of folic acid result in intratubular precipitation of folic acid along the nephron as well as direct cellular injury, causing acute kidney failure. The model is useful because of its simplicity and the fact that, unlike surgical acute ischemia models, many mice can be simultaneously treated. An important weakness in our experience is the extreme variability in the degree of injury of individual mice given identical doses of folic acid. Variation in the extracellular fluid volume, hydration state, and acid base balance would surely have an impact on tendency of folic acid to precipitate in the nephron. The uninjured kidney is populated with interstitial resident macrophages that are thought to play a role in the propagation of injury and release of chemotactic signals that drive further accumulation of inflammatory infiltrate. Peritubular capillaries become filled with inflammatory cells which are thought to contribute to ongoing injury by production of superoxide and cytokines. We find upregulation of CLIC1 in the proximal tubule cells following injury, with a distribution more highly polarized to the apical membrane in cells which have lost their brush border. In contrast to pancreas, we do not see increased nuclear localization of CLIC in injured WT kidney, but rather see less nuclear CLIC1 following injury.

### CLIC1 in macrophages and neutrophils

Macrophages and neutrophils are the two key cell types in most acute inflammatory infiltrates and both are thought to be important sources of superoxide. Both cell types express CLIC1, and in both cells types, CLIC1 is present in both a cytoplasmic distribution and in a peripheral distribution consistent with either the plasma membrane or with the peripheral cytoskeleton. Since superoxide production was decreased in C1KO mice after acute toxic injury of both kidney and pancreas, we examined the superoxide production by these inflammatory cells which normally express CLIC1. Both neutrophils and macrophages are known to generate superoxide in response to stimulation by phorbol ester. Neutrophils generate much more superoxide than macrophages in culture, but the cell type responsible for the bulk of the superoxide production during tissue injury is unclear, and further complicated by reciprocal stimulatory interactions between these two key inflammatory cells by secreted cytokines in the course of an acute inflammatory response. In both cell types, superoxide is produced by the actions of the NOX2 isoform of NADPH oxidase. We found a significant effect of the absence of CLIC1 on superoxide production by both cell types. Unexpectedly, the absence of CLIC1 had the opposite effect on the two cell types, with enhancement of PMA‐induced superoxide production by neutrophils but suppression of PMA‐induced superoxide production by macrophages.

CLIC1 has previously been proposed to have two separate functions in macrophages or macrophage‐like cells. First, microglia, which are CNS analogs of resident tissue macrophages, were shown to have impaired PMA‐stimulated superoxide production after oligonucleotide‐dependent CLIC1 knock down in culture. These investigators found decreased plasma membrane chloride permeability in the CLIC1 knock down cells, and proposed that superoxide production was limited by CLIC1‐mediated plasma membrane chloride permeability. Second, CLIC1 has been proposed to be a component of the membrane of phagocytic vesicles where it supports maximal acidification. Our findings presented here are inconsistent with both prior reports.

First, although we find superoxide production to be impaired in C1KO macrophages, we do not find loss of plasma membrane chloride permeability. In fact, we find increased plasma membrane chloride permeability compared to WT cells that is not further enhanced following PMA in the C1KO cells, and is inhibited by IAA‐94. Furthermore, the redistribution of CLIC1 away from the plasma membrane following PMA strongly suggests that it is unlikely to be responsible for the increased plasma membrane chloride permeability under the same conditions. In fact, the data indicate CLIC1 has the effect of a chloride permeability inhibitor. In the wild type, PMA causes redistribution of CLIC1 away from the membrane, accompanied by activation of an IAA‐inhibitable plasma membrane anion permease; in the absence of CLIC1, this IAA‐inhibitable anion permeability shows constitutive activity that is not further activated by PMA. While we can be confident that this IAA‐inhibited plasma membrane anion permeability is not due to CLIC1 itself, it is not at all certain what protein is responsible. To our knowledge, the only chloride transporters that have been shown to be inhibited by IAA‐94 are the CLIC proteins. Either another member of the CLIC family is present in macrophages and regulated by CLIC1 (e.g., CLIC4 is known to be expressed by macrophages) or a different unidentified macrophage chloride channel can be regulated by CLIC1 and inhibited by IAA‐94.

We did not find co‐localization of CLIC1 with intracellular vesicles in macrophages, either in resting cells, or after labeling phagosomes with zymosan. These results were consistent using either of our CLIC1‐specific antibody preparations. The possible differences with previous reports could be due to mouse strain differences, or differences in specificity of antibodies, or the fact that we did no opsinize the zymosan, but our data do not support enrichment of CLIC1 in phagosomal membranes.

### CLIC1 and the ERM cytoskeleton of macrophages

A growing body of evidence indicates that CLIC family members can interact with cytoskeleton, particularly via ERM components, and furthermore, the CLICs may have some regulatory influence on cytoskeletal organization. The peripheral distribution of CLIC1 and in particular, the abundance of CLIC1 in pseudopodia of cultured macrophages led us to examine a potential role for CLIC1 in regulation of macrophage ERM cytoskeleton. Both ezrin and pERM are present in a peripheral distribution in resting cells, and both redistribute away from the periphery following PMA, with the pERM signal also becoming less intense. We did not discern any systematic difference in distribution of ezrin or pERM between the wild‐type and C1KO mice, either in resting cells or following PMA. Furthermore, we found no difference on western blotting in total pERM in resting cells or in the extent of dephosphorylation of ERM following PMA between WT and C1KO macrophages. Thus, CLIC1 distributes with the cytoskeleton but does not appear to play a major non‐redundant role in regulation of the cytoskeletal distribution or the phosphorylation state of ERM in macrophages. Changes in cytoskeletal dynamics do not appear to be responsible for the altered superoxide production in C1KO macrophages although our methods would not detect subtle changes that could be impactful, and the decreased steady state level of ezrin expression in the C1KO macrophages conceivably could have effects on specific interactions (e.g., with NOX2) without effecting overall cytoskeletal organization or dynamics.

### CLIC1 and subcellular distribution of NOX2

NOX2 is the enzyme responsible for superoxide production by macrophages. This enzyme demonstrates at least two levels of post‐translational regulation. The enzyme consists of a membrane‐embedded component and a peripherally associated component. In un‐activated macrophages, the peripheral component is free in the cytoplasm. Activation of macrophages (e.g., by stimulation with thioglycollate medium) leads to assembly of these two components into a holoenzyme that is present in intracellular membranes but remains inactive. Further stimulation (e.g., by PMA) results in redistribution of this holoenzyme to the plasma membrane where superoxide production can occur. Redistribution to the plasma membrane is necessary for superoxide production by NADPH oxidase in macrophages and our data indicate that this process is blocked in the absence of CLIC1.

Superoxide is generated by NOX2 in neutrophils as well. However, an important difference in regulation of activity in neutrophils and macrophages related to subcellular localization has been long recognized. Neutrophil NOX2 does not require redistribution for activity while macrophage NOX2 does. In this light, the difference in the effect of absence of CLIC1 on superoxide production between macrophages and neutrophils makes some sense, and supports the hypothesis that the key role of CLIC1 in supporting superoxide production in macrophages is through membrane trafficking of the NOX2 following stimulation with PMA. We have not further investigated the increased superoxide production by CLIC1 null neutrophils in culture.

A role for CLIC1 in trafficking of intracellular membranes fits nicely with a large body of prior data: a CLIC is essential for assembly of the intracellular tube that functions as the kidney in C. elegans (Berry et al. 2003). CLIC4 plays an important role in intracellular tubulogenesis in endothelial cells, and in kidney tubulogenesis during development of the nephron (Ulmasov et al. 2009; Chou et al. 2016). Furthermore, in this light, contributions to membrane trafficking rather than mass chloride transport could explain other puzzling observations of CLIC1 functional interaction with CFTR (Edwards 2006), and the role of CLICs in assembly or function of the osteoclast ruffled membrane (Edwards et al. 2006). Still, whether these effects on traffic require function as an anion permease or in some other role is unknown.

## Summary

These experiments demonstrate an important role for CLIC1 in whole animal inflammatory physiology, with significant impact on diseases with human implications. In two independent models of acute tissue injury, the absence of CLIC1 attenuates some aspects of the acute inflammatory injury, and in both, the local production of ROS is suppressed. On the cellular level, CLIC1 is expressed in both of the key cell types in acute inflammatory infiltrates, and in both, activation of superoxide production with PMA is accompanied by redistribution of CLIC1 away from the periphery of the cell. Paradoxically, we find the absence of CLIC1 suppresses superoxide production by macrophages but enhances superoxide production by neutrophils. We examined macrophages in more detail. In resting WT macrophages, CLIC1 is distributed peripherally and similar to the cortical cytoskeleton; chloride permeability and superoxide production are low; NOX2 is excluded from the plasma membrane. Following PMA stimulation of WT cells, both CLIC1 and ERM cytoskeleton redistribute from the periphery and phosphorylation of ERM is decreased; NOX2 subtly shifts its distribution with appearance of some immunostaining at the surface of the cells; superoxide production and chloride permeability increase and the increase in chloride permeability is inhibited by IAA‐94. In the absence of CLIC1, resting macrophages express lower levels of Ezrin, but the distribution and level of phosphorylation of ERM proteins is similar to wild‐type cells; chloride permeability is increased and is IAA sensitive; superoxide production is similar to resting WT cells. Following PMA stimulation of CLIC1 nulls, ERM cytoskeleton redistributes and dephosphorylates similarly to wild type; NOX2 redistribution to the plasma membrane is not detected; neither superoxide production nor chloride permeability changes. The mechanism by which CLIC1 supports superoxide production is not obvious, but two leading hypotheses are disproven: lack of superoxide production cannot be attributed to either lack of plasma membrane chloride permeability, or to a gross failure in cytoskeletal organization or reorganization in response to PMA. Our data show a failure of redistribution of NOX2 in response to PMA; a necessary role for CLIC1 in this redistribution could explain the failure to generate superoxide in response to PMA in the absence of CLIC1. This interpretation is consistent with the divergent effects of the absence of CLIC1 on superoxide production in macrophages and neutrophils.

Our hope that the CLIC1 null mice would easily resolve the many questions surrounding CLIC1 function turns out to have been naive. It seems more questions have been raised than answered. Is the induction of CLIC1 expression in the ductal/tubular epithelial cells following injury of any functional significance in the response to injury? Is the primary source of superoxide the ductal/tubular cells where CLIC1 is prominently overexpressed and if so, does this require CLIC1‐dependent redistribution of NADPH oxidase as in macrophages? Is the absence of the injury‐induced superoxide production in whole injured organs of C1KO mice due to the lack of PMA induced superoxide production by macrophages observed in vitro? What protein is responsible for the IAA‐inhibitable chloride conductance in the CLIC1 null macrophages? Clearly much more needs to be learned before a plausible, comprehensive, internally consistent theory of CLIC1 function can be formulated.

## Conflict of Interest

None declared.
